# The mitochondrial UPR regulator ATF5 promotes intestinal barrier function via control of the satiety response

**DOI:** 10.1016/j.celrep.2022.111789

**Published:** 2022-12-13

**Authors:** Douja Chamseddine, Siraje A. Mahmud, Aundrea K. Westfall, Todd A. Castoe, Rance E. Berg, Mark W. Pellegrino

**Affiliations:** 1 Department of Biology, University of Texas at Arlington, Arlington, TX 76019, USA; 2 Department of Microbiology, Immunology and Genetics, University of North Texas Health Science Center, Fort Worth, TX 76107, USA; 3 Lead contact

## Abstract

Organisms use several strategies to mitigate mitochondrial stress, including the activation of the mitochondrial unfolded protein response (UPR^mt^). The UPR^mt^ in *Caenorhabditis elegans*, regulated by the transcription factor ATFS-1, expands on this recovery program by inducing an antimicrobial response against pathogens that target mitochondrial function. Here, we show that the mammalian ortholog of ATFS-1, ATF5, protects the host during infection with enteric pathogens but, unexpectedly, by maintaining the integrity of the intestinal barrier. Intriguingly, ATF5 supports intestinal barrier function by promoting a satiety response that prevents obesity and associated hyperglycemia. This consequently averts dysregulated glucose metabolism that is detrimental to barrier function. Mechanistically, we show that intestinal ATF5 stimulates the satiety response by transcriptionally regulating the gastrointestinal peptide hormone cholecystokinin, which promotes the secretion of the hormone leptin. We propose that ATF5 protects the host from enteric pathogens by promoting intestinal barrier function through a satiety-response-mediated metabolic control mechanism.

## In brief

Chamseddine et al. demonstrate a role for the mitochondrial UPR regulator ATF5 in protecting the mammalian host during enteric infection. As a part of this defense mechanism, ATF5 promotes intestinal barrier integrity through the stimulation of the satiety response and the prevention of hyperglycemia.

## Graphical abstract

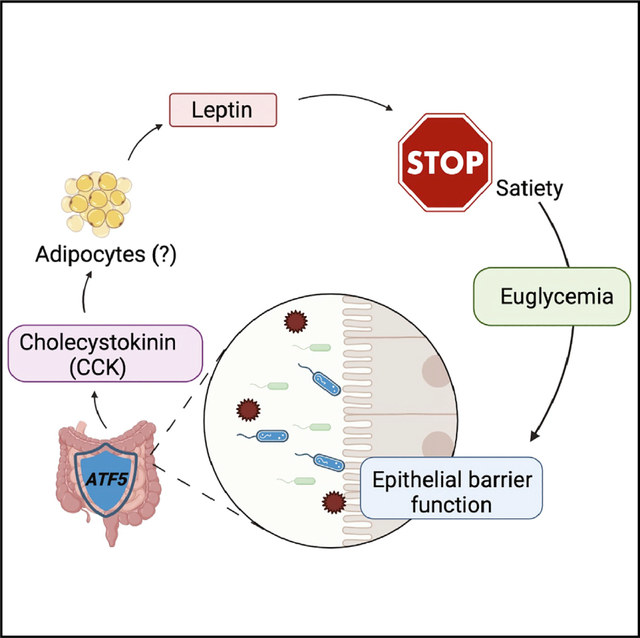

## INTRODUCTION

Mitochondria are essential double-membraned organelles that mediate core cellular functions such as energy production via oxidative phosphorylation and the regulation of programmed cell death. Mitochondria encounter a variety of potential stresses including damage from reactive oxygen species (ROS) that are generated as a by-product of oxidative phosphorylation. They are further challenged by a proteome that is predominantly encoded by the nuclear genome and marginally from their own, thus requiring precise and coordinated expression of genes from each. Finally, and because of their essential function, mitochondria face targeted assault by pathogens such as bacteria and viruses to promote infection.^1,2^ Indeed, many pathogens produce secreted toxins in the form of small molecules or proteins that target the mitochondria. For example, the opportunistic pathogen *Pseudomonas aeruginosa* produces toxins such as cyanide that inhibit oxidative phosphorylation.^3^ In addition, pathogens such as *Vibrio cholerae* and enteropathogenic *Escherichia coli* produce protein virulence factors containing mitochondrial targeting sequences that directly localize to mitochondria to subvert its function.^4,5^

To cope with these many challenges, mitochondria use a diverse set of recovery mechanisms that help to support homeostasis in the face of stress. One such mechanism is the mitochondrial unfolded protein response (UPR^mt^), a retrograde signaling pathway that promotes mitochondrial repair during stress through the regulation of gene expression.^6,7^ A central regulator, the bZIP transcription factor ATFS-1, coordinates the UPR^mt^ in the model organism *Caenorhabditis elegans*.^8,9^ ATFS-1 possesses two cellular localization signals: a mitochondrial targeting sequence and a nuclear localization sequence.^8,10^ The regulation of ATFS-1 is largely dictated by its mitochondrial import efficiency. Under healthy conditions, ATFS-1 is imported into mitochondria and turned over by the protease LONP-1.^8,11^ However, stress impairs mitochondrial import efficiency, preventing the entry of ATFS-1 into mitochondria.^8,12^ Consequently, ATFS-1 accumulates in the cytoplasm and is imported into the nucleus to drive changes in gene expression that support mitochondrial recovery. Consistently, ATFS-1 and the UPR^mt^ are required for animal development during mitochondrial stress because of its critical role in promoting homeostasis.^8^ Other functions have been associated with the UPR^mt^ in *C*. *elegans*, including the regulation of mitochondrial biogenesis^10,13,14^ and lifespan determination.^15–19^ In addition to mitochondrial recovery, the UPR^mt^ also functions in protecting the host during infection from pathogenic bacteria such as *P*. *aeruginosa*^20,21^ that target mitochondrial function.^20,22^ Interestingly, the UPR^mt^ not only promotes the expression of genes associated with mitochondrial recovery, but also those with functions in innate immunity and pathogen defense. As such, the UPR^mt^ is both required and sufficient to protect the *C*. *elegans* host during infection.^16,18–21,23^

The bZIP transcription ATF5 is the likely mammalian ortholog of ATFS-1, because it can functionally replace ATFS-1 in *C*. *elegans* to drive the UPR^mt 24^. ATF5 also possesses a mitochondrial targeting sequence and is thought to be similarly regulated by mitochondrial import efficiency to activate the UPR^mt 24^. Much like ATFS-1, ATF5 is required for cell survival *in vitro* in the presence of mitochondrial stress,^24^ but relatively less is known regarding its physiological function in mammals. Recently, ATF5 and the UPR^mt^ were shown to have cardioprotective properties during ischemia-reperfusion injury.^25^ However, evidence of additional roles of ATF5/UPR^mt^ outside this model are lacking. Notably, whether ATF5 and the UPR^mt^ possess a similar function as *C*. *elegans* ATFS-1 in protecting the host during pathogen infection has yet to be explored. Here, we provide evidence that ATF5/UPR^mt^ defends the mammalian host during infection by enteric pathogens. Intestinal ATF5 protects the host during enteric infection by promoting intestinal barrier function, thus preventing the infiltration of microbes and toxins into underlying tissues. Interestingly, ATF5 supports intestinal barrier function by preventing excessive glycolytic flux. This is achieved by averting hyperglycemia through stimulation of a leptin-mediated satiety response. We show that intestinal ATF5 controls leptin levels and the satiety response through regulation of the gut-derived hormone, cholecystokinin. Together, our data illustrate a function for the UPR^mt^ regulator ATF5 in promoting intestinal barrier function through a metabolically mediated mechanism involving appetite control.

## RESULTS

### ATF5 protects the host during enteric infection

We sought to explore the function of ATF5 and the mammalian UPR^mt^ in the intestinal epithelium during enteric infection. As a first step, we determined the expression pattern of ATF5 in the mouse intestine. ATF5 was detectable in all intestinal segments, including the duodenum, jejunum, ileum, and colon (Figure S1A). A greater level of ATF5 was observed in the jejunum relative to other regions. Using immunohistochemistry, ATF5 expression was detected in the intestinal villi and crypts, enterocytes, Paneth cells, lamina propria, zymogen granules, submucosal glands, and muscularis (Figure S1B).

**Figure 1.**
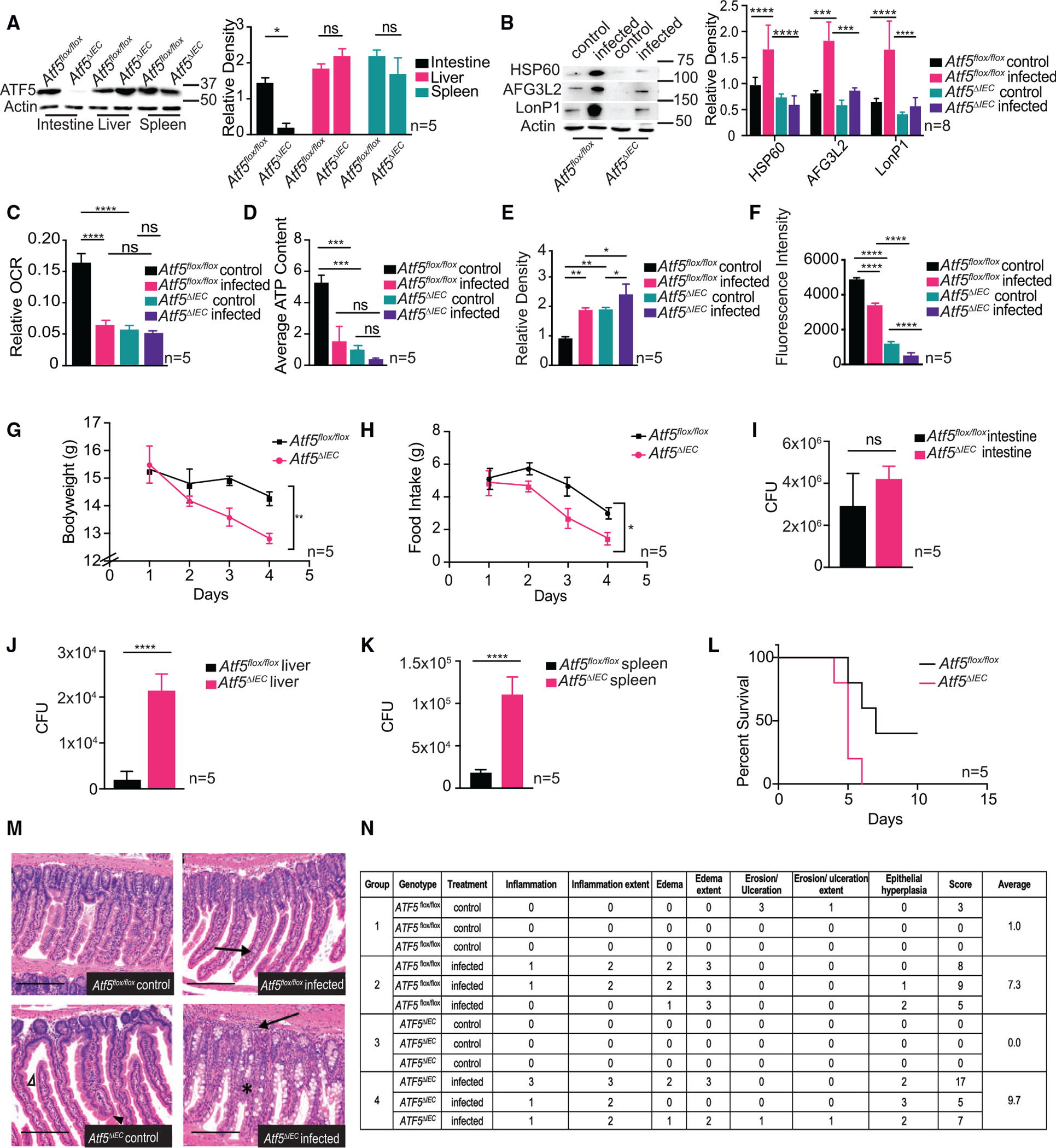
ATF5 protects the host during enteric infection (A) Immunoblot analysis and quantification of ATF5 protein levels in the intestine, liver, and spleens of *Atf5*^*flox/flox*^ and *Atf5*^*ΔIEC*^ mice. Actin was used as a loading control. Data represent mean ± standard error of the mean (n = 5; ns, non-significant, *p < 0.05 using the Student’s t test). (B) Immunoblot analysis and quantification of UPR^mt^-related proteins in the presence or absence of *Salmonella* infection in *Atf5*^*flox/flox*^ and *Atf5*^*ΔIEC*^ mice. Actin was used as a loading control. Data represent mean ± standard error of the mean (n = 8; ***p < 0.001,****p < 0.0001 using the Student’s t test). (C–E) Oxygen consumption rate (OCR) (C), ATP production (D), and oxidative damage (E) from small intestine samples of *Atf5*^*flox/flox*^ and *Atf5*^*IEC*^ mice in the presence or absence of *Salmonella* infection. Data represent mean ± standard error of the mean (n = 5; ns, non-significant, *p < 0.05,**p < 0.001, ***p < 0.001,****p < 0.0001 using the Student’s t test). (F) Mitochondrial membrane potential quantification using TMRE from small intestine samples of *Atf5*^*flox/flox*^ and *Atf5*^*ΔIEC*^ mice in the presence or absence of *Salmonella* infection. Data represent mean ± standard error of the mean (n = 5; ****p < 0.0001 using the Student’s t test). (G and H) Changes in bodyweight (G) and feeding (H) of *Atf5*^*flox/flox*^ and *Atf5*^*ΔIEC*^ mice during challenge with *Salmonella*. Data represent mean ± standard error of the mean (n = 5; *p < 0.05, **p < 0.001 using the Student’s t test). (I–K) Colony-forming units (CFU) of intestine (I), liver (J) and spleen (K) samples from *Salmonella* infected *Atf5*^*flox/flox*^ and *Atf5*^*ΔIEC*^ mice. (n = 5; ns, non-significant, ****p < 0.0001 using the Student’s t test). (L) Survival of *Atf5*^*flox/flox*^ and *Atf5*^*ΔIEC*^ mice during challenge with *Salmonella* (n = 5). See Table S1 for all statistics pertaining to survival analysis. (M and N) Representative histological analysis (M) and pathology score table (N) of small intestine tissue sections from *Atf5*^*flox/flox*^ and *Atf5*^*ΔIEC*^ mice during challenge with *Salmonella* (closed arrowhead represents reduced villi height, open arrowhead represents increased spacing between intestinal crypts, arrow and asterisk represent neutrophilic inflammation and villous fusion, respectively; n = 3). Scale bars, 200 μm.

We next explored the relationship of ATF5 and host protection during enteric infection using the pathogen *Salmonella enterica* subsp. *enterica* (Serovar Typhimurium), hereafter referred to as *Salmonella*. We first examined whether ATF5 promotes mitochondrial recovery during infection. Whole-animal *Atf5* knockout in mice results in neonatal lethality from a defect in olfactory neuron differentiation that prevents competitive suckling at birth. The mice that escape this neonatal death are able to develop but weigh less than their wild-type siblings.^26^ To circumvent this issue, we created a tissue-specific *Atf5* knockout in the intestine. We bred *Atf5*^*flox/flox*^ mice with mice expressing Cre recombinase under the control of the intestinal epithelium-specific promoter *villin*. We confirmed that intestinal ATF5 expression was minimally detectable in *Atf5*^*flox/flox*^ homozygous, *villin-Cre* heterozygous mice (*Atf5*^*ΔIEC*^) (Figure 1A). We then measured the expression of various markers of the UPR^mt^, including the mitochondrial chaperone heat shock protein 60 and the mitochondrial proteases AFG3L2 and LONP1,^24,27,28^ in the presence or absence of *Salmonella* infection. Mice challenged with *Salmonella* infection displayed higher expression of all UPR^mt^ markers compared with uninfected control mice, which were suppressed in *Atf5*^*ΔIEC*^ mice (Figure 1B). Therefore, *Salmonella* enteric infection results in an ATF5-dependent UPR^mt^. We observed that many mitochondrial functions that were impaired during *Salmonella* infection were further compromised in the absence of ATF5. These dysfunctions included a reduced oxygen consumption rate (Figure 1C), lower adenosine triphosphate (ATP) production (Figure 1D), increased oxidative damage (Figure 1E), and reduced mitochondrial membrane potential (Figure 1F). Interestingly, mitochondrial function was also reduced in *Atf5*^*ΔIEC*^ mice in the absence of infection (Figures 1C–1F), suggesting that ATF5 possesses activity in the intestine under physiological conditions. Together, our data support a function of ATF5 in mediating mitochondrial recovery in the intestinal epithelium.

We next assessed the susceptibility of *Atf5*^*ΔIEC*^ mice to enteric infection with *Salmonella*. We observed that decreases in bodyweight and feeding resulting from *Salmonella* infection were exacerbated in infected *Atf5*^*ΔIEC*^ mice (Figures 1G and 1H). Furthermore, while *Salmonella* accumulation in the intestine was unaltered in *Atf5*^*ΔIEC*^ mice (Figure 1I), we observed higher levels of *Salmonella* that had disseminated to distal organs such as the liver and spleen in these mutant animals (Figures 1J and 1K). In addition, *Atf5*^*ΔIEC*^ mice infected with *Salmonella* displayed a pronounced rolling behavior as the infection proceeded that was not observed in infected *Atf5*^*flox/flox*^ control animals (Videos S1 and S2). This type of rolling behavior has previously been observed when *Salmonella* disseminates through the bloodstream to the brain.^29^ Importantly, *Atf5*^*ΔIEC*^ mice succumbed to *Salmonella* infection at a faster rate than control animals (Figure 1L), suggesting that ATF5 plays a pivotal protective role in the intestinal epithelium to protect the host during enteric infection. Consistent with this finding, we detected more pronounced damage to the intestinal epithelia during infection with *Salmonella* in the absence of ATF5 (Figures 1M and 1N). More extensive pathologic findings were observed in the jejunum compared with the duodenum and ileum, which is consistent with our observation of higher ATF5 levels in this particular intestinal segment (Figure S1A). Our findings included increased numbers of neutrophils present within the lamina propria that were often diffuse in appearance. Epithelial hyperplasia within the crypt epithelium was also observed, characterized by lengthening of the crypts, crowding of epithelial cells, and cytoplasmic basophilia. Goblet cell hyperplasia was also observed in *Atf5*^*ΔIEC*^-infected mice. Remarkably, we also detected some modest abnormalities to gut epithelial integrity in *Atf5*^*ΔIEC*^ mice even in the absence of infection, including decreased villi height and increased depth of the intestinal crypts (Figures 1M and 1N), suggesting that ATF5 promotes gut epithelial homeostasis even under basal conditions.

Because our study used male-exclusive cohorts, we tested whether the susceptibility of *Atf5*^*ΔIEC*^ mice to infection exhibited sexual dimorphism. We found that female *Atf5*^*ΔIEC*^ mice were similarly hypersusceptible to *Salmonella* challenge, demonstrating reduced bodyweight and feeding behavior during the infection period (Figures S2A and S2B), as well as decreased survival rates (Figure S2C). Therefore, the function of ATF5 in protecting the host during enteric infection is not sex-specific.

To determine whether the ability of ATF5 to protect the host from enteric infection was specific to *Salmonella*, we challenged mice with *Citrobacter rodentium*, a murine mucosal bacterial pathogen used as a model for human enteric infections.^30^ Similar to our findings using *Salmonella*, *Atf5*^*ΔIEC*^ mice challenged with *C*. *rodentium* displayed greater weight loss and decreased feeding behavior relative to *Atf5*^*flox/flox*^ animals (Figures S3A and S3B). Furthermore, we observed higher levels of *C*. *rodentium* in the distal organs of *ATF5*^*ΔIEC*^ mice (Figures S3C and S3D). Finally, *ATF5*^*ΔIEC*^ mice succumbed faster to *C*. *rodentium* infection compared with control mice (Figure S3E). These results suggest that ATF5 likely plays a broad role in protecting the host during enteric infection.

### ATF5 prevents enteric pathogen dissemination by promoting intestinal barrier function

**Figure 2.**
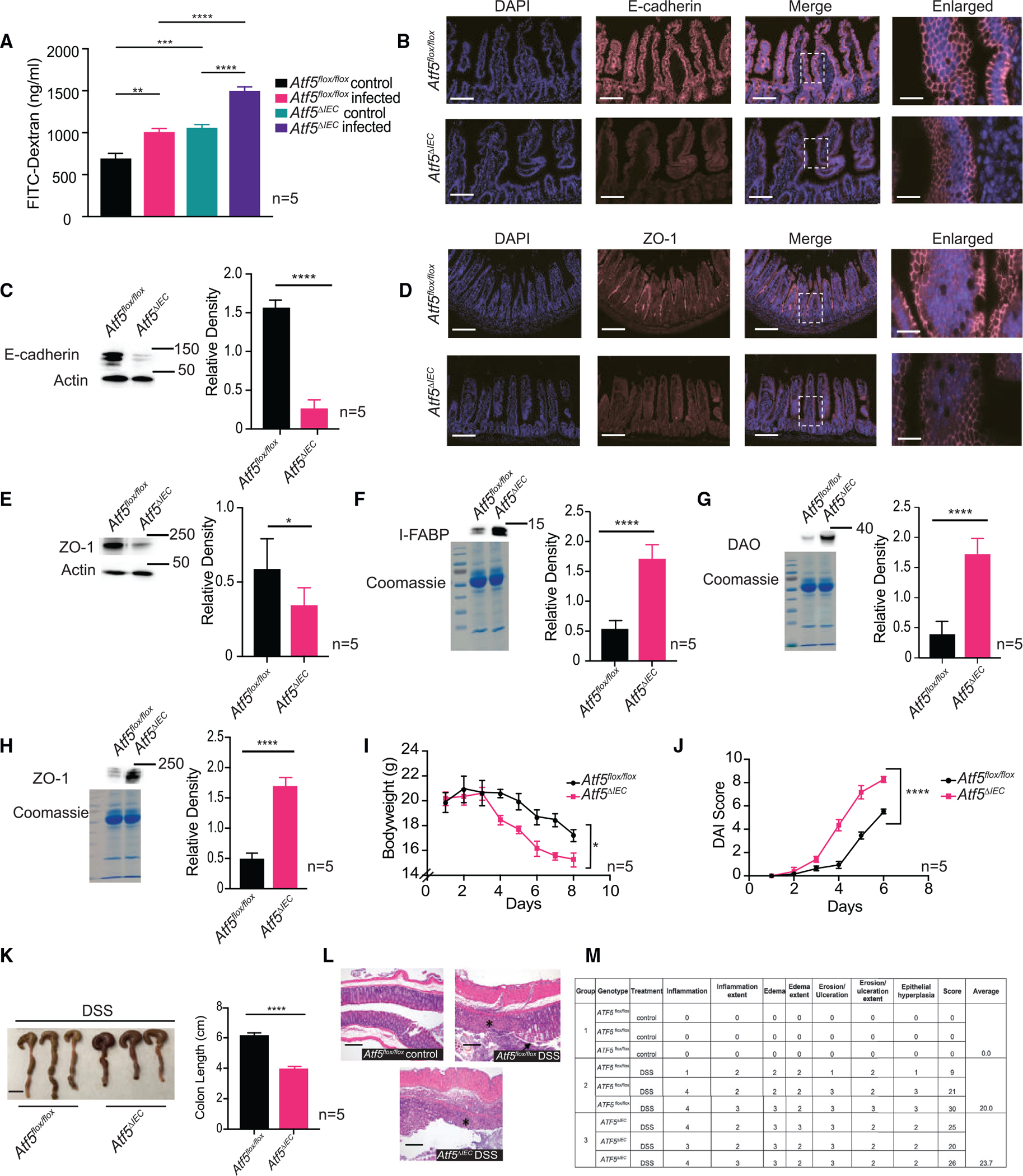
ATF5 prevents enteric pathogen dissemination by promoting intestinal barrier function (A) Serum FITC-dextran levels in *Atf5*^*flox/flox*^ and *Atf5*^*ΔIEC*^ mice. Data represent mean ± standard error of the mean (n = 5; **p < 0.01, ***p < 0.001, ****p < 0.0001 using the Student’s t test). (B) Immunohistochemistry of *Atf5*^*flox/flox*^ and *Atf5*^*ΔIEC*^ intestinal samples using anti-E-cadherin antibody and DAPI co-stain (n = 3). Representative images shown (boxed region denotes enlarged area). Scale bars indicate 100 μm (wide images) or 10 μm (enlarged images). (C) Immunoblot analysis and quantification of E-cadherin protein levels in *Atf5*^*flox/flox*^ and *Atf5*^*ΔIEC*^ mice. Actin was used as a loading control. Data represent mean ± standard error of the mean (n = 5; ****p < 0.0001 using the Student’s t test). (D) Immunohistochemistry of *Atf5*^*flox/flox*^ and *Atf5*^*ΔIEC*^ intestinal samples using anti-ZO-1 antibody and DAPI co-stain (n = 3). Representative images shown (boxed region denotes enlarged area). Scale bars indicate 100 μm (wide images) or 10 μm (enlarged images). (E) Immunoblot analysis and quantification of ZO-1 protein levels in *Atf5*^*flox/flox*^ and *Atf5*^*ΔIEC*^ mice. Actin was used as a loading control. Data represent mean ± standard error of the mean (n = 5; *p < 0.05 using the Student’s t test). (F–H) Immunoblot analysis and quantification of I-FABP (F), DAO (G), and ZO-1 (H) serum levels in *Atf5*^*flox/flox*^ and *Atf5*^*ΔIEC*^ intestinal samples. Coomassie stained SDS-PAGE gels are shown as loading controls (n = 5; ****p < 0.0001 using the Student’s t test). (I and J). Bodyweight (I) and DAI scores (J) of *Atf5*^*flox/flox*^ and *Atf5*^*ΔIEC*^ mice exposed to 4% DSS. Data represent mean ± standard error of the mean (n = 5; *p < 0.05, ****p < 0.0001 using the Student’s t test). (K) Representative image and quantification of colon lengths in *Atf5*^*flox/flox*^ and *Atf5*^*ΔIEC*^ mice exposed to DSS (n = 5). Data represent mean ± standard error of the mean (n = 5; ****p < 0.0001 using the Student’s t test). Scale bar, 1 cm. (L and M) Representative histological analysis and pathology score table of colon tissue sections from *Atf5*^*flox/flox*^ and *Atf5*^*ΔIEC*^ mice exposed to DSS (arrow and asterisk represent epithelial hyperplasia and extensive ulceration and inflammation, respectively; n = 3). Scale bars, 200 μm.

We were intrigued that *Atf5*^*ΔIEC*^ mice displayed abnormalities in intestinal structure even in the absence of infection, suggesting that ATF5 promotes intestinal homeostasis under physiological conditions. Indeed, the observations of altered villi and crypt morphology, as well as increased pathogen dissemination to distal organs in *ATF5*^*ΔIEC*^ mice, suggested a role for ATF5 in promoting intestinal barrier function.^31,32^ The mammalian intestinal epithelium is composed of a complex set of junctional proteins that prevent microbes and other foreign materials of the intestinal lumen from reaching the internal milieu, and we were motivated to explore the role of ATF5 in maintaining the integrity of this barrier. As a first step, we quantified intestinal barrier integrity in the presence or absence of ATF5 by first using fluorescein isothiocyanate (FITC)-dextran, a fluorescently labeled probe that can be used to measure intestinal permeability.^33^ Consistent with our histology findings showing a disrupted intestinal barrier function, we observed higher levels of FITC-dextran in the serum of male and female *Atf5*^*ΔIEC*^ mice compared with control animals (Figures 2A and S2D). A perturbed epithelial barrier integrity was also observed in *Atf5*^*flox/flox*^ control animals infected with *Salmonella* and exacerbated in *Atf5*^*ΔIEC*^-infected mice (Figure 2A). In addition, we observed reduced levels of epithelial junctional proteins including the adherens junctional protein E-cadherin (Figures 2B and 2C) and the tight junction protein zonula occludens (ZO-1) (Figures 2D and 2E) in uninfected *Atf5*^*ΔIEC*^ intestinal samples, two markers of epithelial barrier integrity.^34–36^ Furthermore, E-cadherin was more punctate in appearance in *Atf5*^*ΔIEC*^ mice compared with control animals, which is indicative of a reduced cell-cell boundary structure.^37^ To further assess intestinal barrier function, we quantified serum biomarkers of intestinal permeability, diamine oxidase (DAO)^38^ and intestinal fatty acid binding protein (I-FABP),^39,40^ both of which were elevated in *Atf5*^*ΔIEC*^ serum (Figures 2F and 2G). We also detected higher levels of ZO-1 in serum samples from *Atf5*^*ΔIEC*^ mice (Figure 2H), consistent with a loss of epithelial barrier integrity.^41^

We next examined the role of ATF5 in promoting intestinal barrier function by measuring animal responses to dextran sulfate sodium (DSS), a chemical used to model colitis in rodents.^42^ We observed a greater loss of bodyweight and an increased disease activity index (DAI) score (a measure of colitis progression^43^) in *Atf5*^*ΔIEC*^ mice after DSS administration (Figures 2I and 2J). Furthermore, colons of *Atf5*^*ΔIEC*^ mice were significantly shorter than those of *Atf5*^*flox/flox*^ mice when each were treated with DSS (Figure 2K), indicative of colitis and inflammation.^44^ Intestinal epithelial tissue sections of mice treated with DSS displayed greater colitis scores compared with *Atf5*^*flox/flox*^ animals, with extensive inflammation, ulceration, and epithelial hyperplasia, which were more severe in *Atf5*^*ΔIEC*^-treated mice (Figures 2L and 2M). These findings support an integral role of ATF5 in promoting intestinal barrier function.

**Figure 3.**
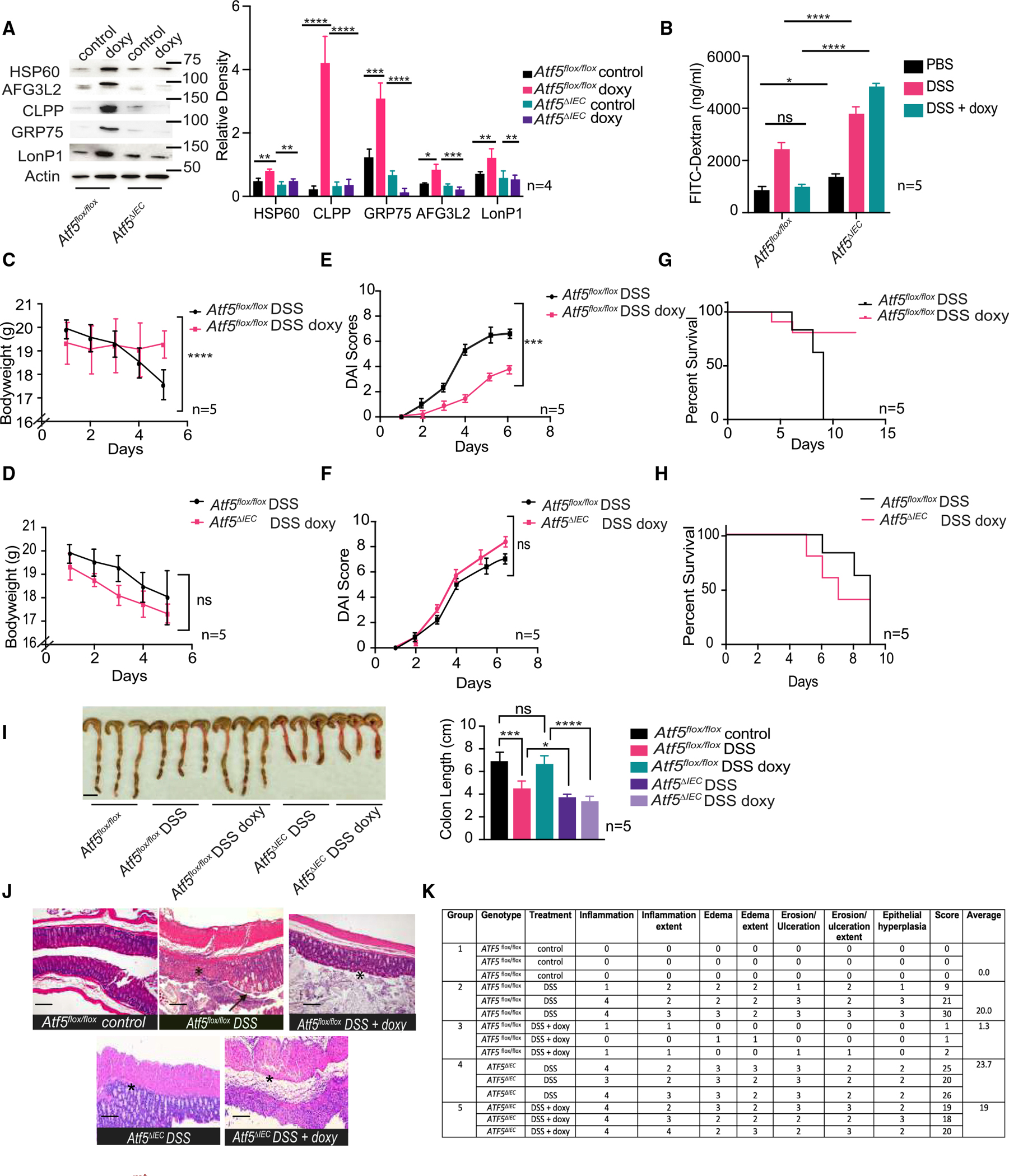
The UPR^mt^ protects against colitis in an ATF5-dependent manner (A) Immunoblot analysis and quantification of UPR^mt^-related proteins in the presence or absence of 1.5g/L doxycycline in *Atf5*^*flox/flox*^ and *Atf5*^*ΔIEC*^ mice. Actin was used as a loading control. Data represent mean ± standard error of the mean (n = 4; *p < 0.05, **p < 0.01, ***p < 0.001, ****p < 0.0001 using the Student’s t test). (B) Serum FITC-dextran levels in *Atf5*^*flox/flox*^ and *Atf5*^*ΔIEC*^ mice pre-treated with 1.5 g/L doxycycline and subsequently exposed to 4% DSS or mock control. Data represent mean ± standard error of the mean (n = 5; ns, non-significant, *p < 0.05, ****p < 0.0001 using the Student’s t test). (C and D) Bodyweight of *Atf5*^*flox/flox*^ (C) and *Atf5*^*ΔIEC*^ (D) mice pre-treated with 1.5 g/L doxycycline and subsequently exposed to 4% DSS. Data represent mean ± standard error of the mean (n = 5; ns, non-significant, ****p < 0.0001 by the Student’s t test). (E and F) DAI scores of *Atf5*^*flox/flox*^ (E) and *Atf5*^*ΔIEC*^ (F) mice pre-treated with 1.5 g/L doxycycline and subsequently exposed to 4% DSS or mock control. Data represent mean ± standard error of the mean (n = 5; ns, non-significant, ***p < 0.001 by the Student’s t test). (G and H) Survival of *Atf5*^*flox/flox*^ (G) and *Atf5*^*ΔIEC*^ (H) mice pre-treated with 1.5 g/L doxycycline and subsequently exposed to 4% DSS or mock control (n = 5). See Table S1 for all statistics pertaining to survival analysis. (I) Representative image and quantification of colon lengths in *Atf5*^*flox/flox*^ and *Atf5*^*ΔIEC*^ mice exposed to 4% DSS, in the presence or absence of 1.5 g/L doxycycline pre-treatment. Data represent mean ± standard error of the mean (n = 5; ns, non-significant, *p < 0.05, ***p < 0.001, ****p < 0.0001 using the Student’s t test). Scale bar, 1 cm. (J and K) Representative histological analysis and pathology score table of small intestine tissue sections from *Atf5*^*flox/flox*^ and *Atf5*^*ΔIEC*^ mice pre-treated with 1.5 g/L doxycycline, followed by exposure to 4% DSS (arrow and asterisk represent epithelial hyperplasia and extensive ulceration and inflammation, respectively; n = 3). Scale bars, 200 μm.

Knowing that ATF5 was required to maintain intestinal barrier integrity, we next explored whether priming animals with the UPR^mt^ could protect against subsequent damage to the gut epithelial barrier. This was accomplished by first inducing ATF5 expression and then subsequently measuring the response of these primed animals to DSS. We treated animals with the antibiotic doxycycline, previously shown to stimulate the UPR^mt^ as a result of mitonuclear imbalances.^25,45,46^ As expected, doxycycline induced the expression of UPR^mt^ targets in the intestine of control animals in an ATF5-dependent manner (Figure 3A). We observed a striking improvement in intestinal barrier function in doxycycline-treated mice that was suppressed in *ATF5*^*ΔIEC*^ mice (Figure 3B). Treatment of *ATF5*^*flox/flox*^ animals with doxycycline also significantly improved weight loss, DAI scores, and host survival during exposure with DSS, but not in *Atf5*^*ΔIEC*^ animals (Figures 3C–3H). Furthermore, we found that the decrease in colon length caused by DSS treatment was suppressed in UPR^mt^-primed animals in an ATF5-dependent manner (Figure 3I). Histologically, animals primed for the UPR^mt^ displayed improved barrier function in the presence of DSS, with only very mild inflammation, edema, and epithelial erosion (Figures 3J and 3K). These protective effects were lost in the absence of ATF5, whereby DSS-exposed *Atf5*^*ΔIEC*^ animals primed for the UPR^mt^ displayed characteristic features of colitis including shortened villi, extensive inflammation (including that of the submucosa), edema, and secretion of inflammatory cells (Figures 3J and 3K). These results suggest that the UPR^mt^ maintains intestinal barrier function in an ATF5-dependent manner.

We also explored whether ATF5 regulated other parameters related to intestinal homeostasis, including differentiation, proliferation, and survival. We measured the expression of Lgr5 and Sox9 as a proxy for intestinal differentiation, which are markers of the crypt stem cell niche that gives rise to all small intestine epithelial cell types.^47,48^ Both Lgr5 and Sox9 were not reduced in *Atf5*^*ΔIEC*^ mice (Figures S4A and S4B), suggesting that ATF5 is not involved in the differentiation of intestinal enterocytes. However, we did observe that Lgr5 and Sox9 expression levels were increased in *Atf5*^*ΔIEC*^ mice (Figures S4A and S4B). Indeed, Lgr5 and Sox9 are regulated transcriptionally by various pathways,^49–51^ as well as post-translationally by a protein turnover mechanism,^52^ either of which may be mediated by ATF5. It is, therefore, possible that ATF5 acts as a negative regulator for both of these targets through an as-yet unidentified mechanism. We also measured intestinal cell proliferation using bromodeoxyuridine (BrdU) staining, which was unaltered in the absence of ATF5 (Figure S4C). Similarly, measures of apoptosis using TUNEL staining of intestinal epithelial cells in the presence or absence of ATF5 indicated no change in control versus *ATF5*^*ΔIEC*^ animals (Figure S4D). Together, ATF5 is required for barrier function but does not seem to play a role in epithelial differentiation.

### ATF5 promotes intestinal barrier integrity via regulation of cholecystokinin/leptin-mediated appetite control

**Figure 4.**
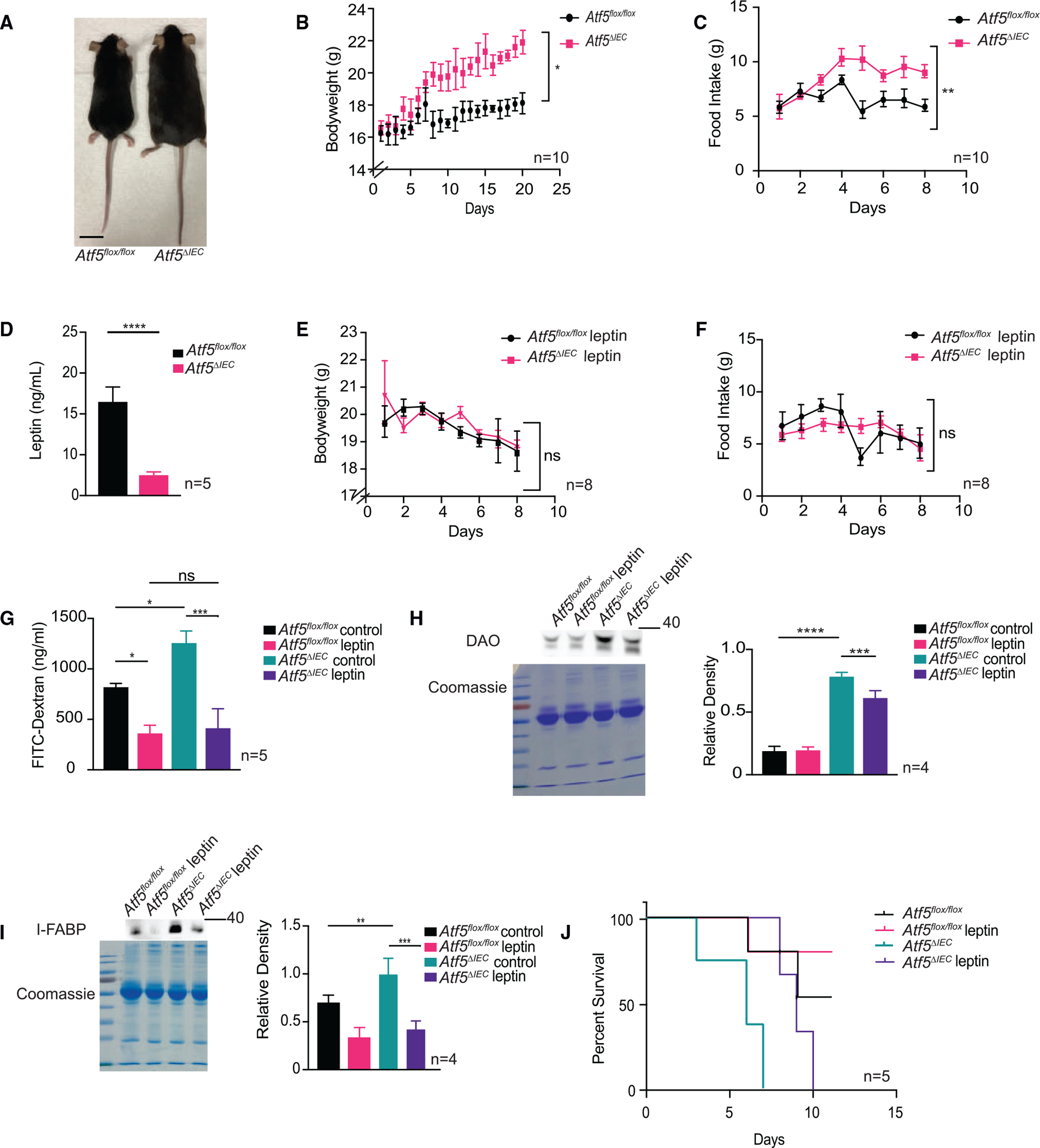
ATF5 promotes intestinal barrier homeostasis via regulation of leptin signaling (A) Representative image of *Atf5*^*flox/flox*^ and *Atf5*^*ΔIEC*^ mice (*n = 4*). Scale bar indicates 1.5 cm. (B and C) Bodyweight (B) and feeding behavior (C) of *Atf5*^*flox/flox*^ and *Atf5*^*ΔIEC*^ mice fed a standard diet. Day 0 represents start of experiment at 6 weeks of age. Data represent mean ± standard error of the mean (n = 10; *p < 0.05, **p < 0.01 using the Student’s t test). (D) Serum leptin levels in 6-week-old *Atf5*^*flox/flox*^ and *Atf5*^*ΔIEC*^ mice. Data represent mean ± standard error of the mean (n = 5; ****p < 0.0001 using the Student’s t test). (E and F) Bodyweight (E) and feeding behavior (F) of *Atf5*^*flox/flox*^ and *Atf5*^*ΔIEC*^ mice intraperitoneally injected with leptin and fed a standard diet. Day 0 represents start of experiment at 6 weeks of age. Data represent mean ± standard error of the mean (n = 8; ns, non-significant using the Student’s t test). (G) Serum FITC-dextran levels in *Atf5*^*flox/flox*^ and *Atf5*^*ΔIEC*^ mice intraperitoneally injected with leptin. Data represent mean ± standard error of the mean (n = 5; ns, non-significant, *p < 0.05, ***p < .001 using the Student’s t test). (H and I) Immunoblot analysis and quantification of DAO (H) and I-FABP (I) serum protein levels in *Atf5*^*flox/flox*^ and *Atf5*^*ΔIEC*^ mice intraperitoneally injected with leptin. Coomassie stained SDS-PAGE gels are shown as loading controls. Data represent mean ± standard error of the mean (n = 4; **p < 0.01, ***p < 0.001, ****p < 0.0001 using the Student’s t test). (J) Survival of *Atf5*^*flox/flox*^*, Atf5*^*ΔIEC*^, and leptin-treated *Atf5*^*ΔIEC*^ mice challenged with *Salmonella* (n = 5). See Table S1 for all statistics pertaining to survival analysis.

We were interested in uncovering the mechanism of intestinal barrier regulation by ATF5. Over the course of this study, we observed that male and female *Atf5*^*ΔIEC*^ mice gained more bodyweight over time compared with *Atf5*^*flox/flox*^ control mice when fed a standard chow diet (Figures 4A, 4B and S2E). In addition, *Atf5*^*ΔIEC*^ mice tended to eat more compared with their control littermates (Figures 4C and S2F). Given the relationships between obesity and the integrity of the intestinal barrier,^53–56^ we were motivated to explore the relationships between ATF5 and regulation of weight gain and feeding behavior. Based on this similarity, we hypothesized that leptin hormone signaling, one of the predominant pathways controlling satiety (i.e., the response of feeling well-fed after feeding), might be acting downstream of ATF5. Animals that are deficient in leptin or leptin signaling have increased appetites and become obese,^57–59^ similar to *Atf5*^*ΔIEC*^ mice. We first measured serum levels of leptin in the presence or absence of ATF5 and observed a strikingly low level of this hormone in *Atf5*^*ΔIEC*^ animals relative to *Atf5*^*flox/flox*^ controls (Figure 4D). To explore whether reduced leptin availability was the cause of the abnormal feeding behavior and weight gain in *Atf5*^*ΔIEC*^ animals, we re-introduced leptin into control and mutant animals via intraperitoneal injection. Indeed, we found that treatment of *Atf5*^*ΔIEC*^ mice with leptin restored wild-type satiety behavior, as demonstrated by their normal feeding behavior and body mass (Figures 4E and 4F). Therefore, intestinal ATF5 regulates animal bodyweight via control of leptin availability and the satiety response.

We next explored whether the impaired intestinal barrier function of *Atf5*^*ΔIEC*^ mice was due to perturbed leptin signaling. Using the FITC-dextran assay, we found that intraperitoneal injection of leptin into *Atf5*^*ΔIEC*^ mice restored intestinal barrier function to levels similar to those in *Atf5*^*flox/flox*^ control animals (Figure 4G). In addition, serum biomarkers of intestinal permeability DAO and I-FABP were reduced in *Atf5* ^*ΔIEC*^ animals after the administration of leptin (Figures 4H and 4I). These findings strongly suggest that ATF5 protects the host from enteric infection by promoting intestinal barrier function via the regulation of leptin signaling. As validation, we examined whether the reintroduction of leptin could improve the susceptibility of *Atf5*^*ΔIEC*^ animals to *Salmonella*, and found that, indeed, the survival of *Atf5*^*ΔIEC*^ mice intraperitoneally injected with leptin was comparable with that of *Atf5*^*flox/flox*^ control animals when infected with *Salmonella* (Figure 4J).

How does intestinal ATF5 regulate serum leptin levels? Leptin is secreted mainly from the adipose tissue,^60^ in addition to the gastric mucosa, placenta, muscle, and brain.^61^ To our knowledge, there have been no reports of intestinal leptin regulating the satiety response.

**Figure 5.**
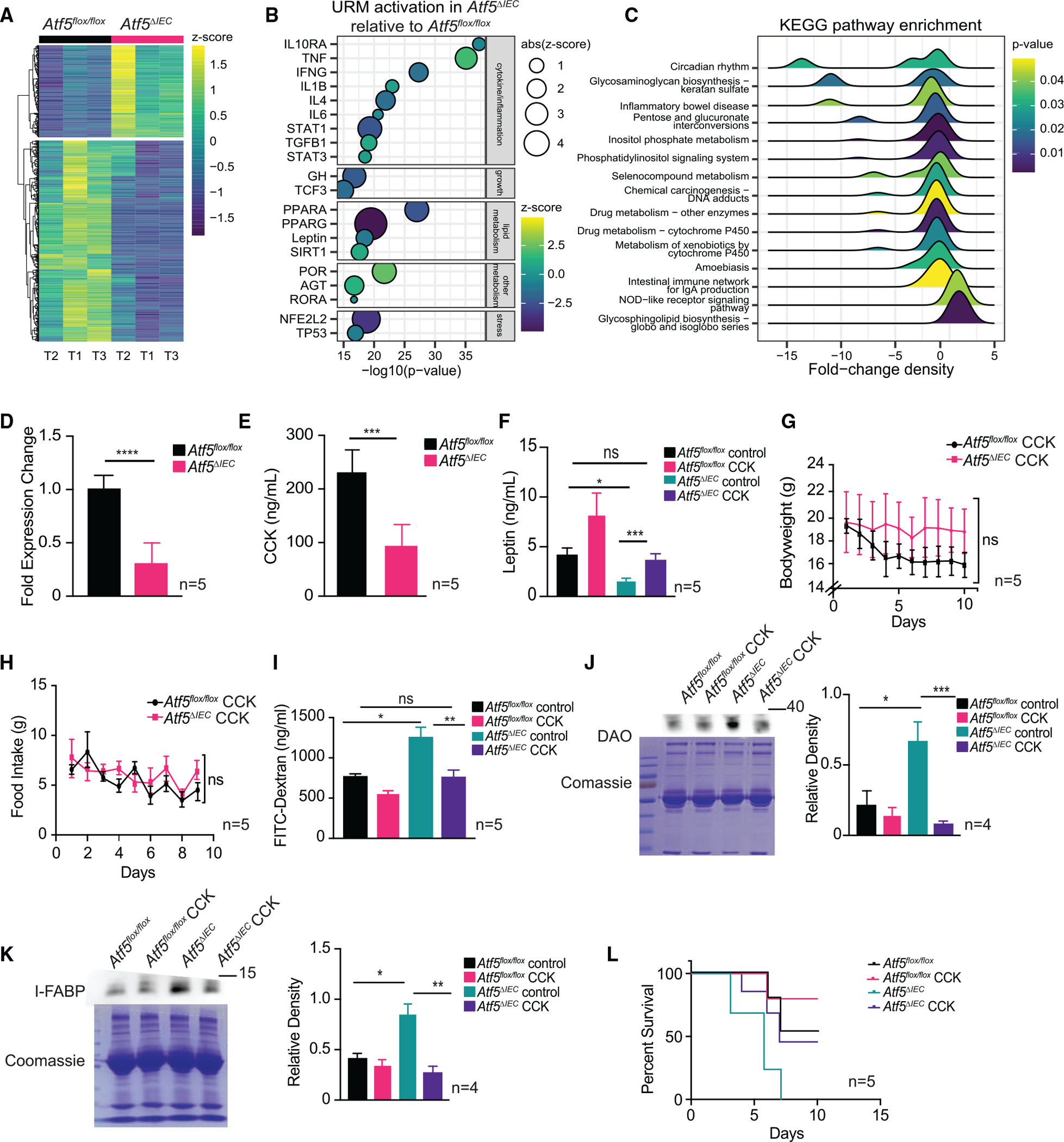
ATF5 controls leptin-mediated satiety response and intestinal barrier function through regulation of CCK (A) Heatmap of differentially expressed genes in intestinal tissues of *Atf5*^*ΔIEC*^ mice compared with *Atf5*^*flox/flox*^ controls, including 269 up and 588 downregulated genes (n = 3). (B) Top 20 URMs predicted based on an ingenuity pathway analysis of genes differentially expressed in intestinal tissues, showing the inferred activation of URMs in *Atf5*^*ΔIEC*^ mice relative to *Atf5*^*flox/flox*^ controls. Z-scores for URM activation are based on observed patterns of gene expression for genes downstream of respective URMs, where the magnitude of the *Z* score represents evidence for differential activation in *Atf5*^*ΔIEC*^ mice relative *Atf5*^*flox/flox*^ controls, and the sign of the *Z* score indicates the direction of activation (positive) or repression (negative) in *Atf5*^*ΔIEC*^ mice relative to *Atf5*^*flox/flox*^ controls (n = 3). (C) Top 15 enriched KEGG pathways from differentially expressed genes in *Atf5*^*ΔIEC*^ mice compared with *Atf5*^*flox/flox*^ controls, with distributions of fold change for differentially expressed genes within each KEGG pathway. (D) *Cck* transcript levels measured by qRT-PCR in *Atf5*^*flox/flox*^ and *Atf5*^*ΔIEC*^ mice. Data represent mean ± standard error of the mean (n = 5; ****p < 0.0001 using the Student’s t test). (E) Serum CCK levels in *Atf5*^*flox/flox*^ and *Atf5*^*ΔIEC*^ mice. Data represent mean ± standard error of the mean (n = 5; ***p < 0.001 using the Student’s t test). (F) Serum leptin levels in *Atf5*^*flox/flox*^ and *Atf5*^*ΔIEC*^ mice, with or without intraperitoneal CCK injection. Data represent mean ± standard error of the mean (n = 5; ns, non-significant, *p < 0.05, ***p < 0.001 using the Student’s t test). (G and H) Bodyweight (G) and feeding behavior (H) of *Atf5*^*flox/flox*^ and *Atf5*^*ΔIEC*^ mice intraperitoneally injected with CCK. Day 0 represents start of experiment at 6 weeks of age. Data represent mean ± standard error of the mean (n = 5; ns, non-significant using the Student’s t test). (I) Serum FITC-dextran levels in *Atf5*^*flox/flox*^ and *Atf5*^*ΔIEC*^ mice, with or without intraperitoneal CCK injection. Data represent mean ± standard error of the mean (n = 5; ns, non-significant, *p < 0.05, **p < 0.01 using the Student’s t test). (J and K) Immunoblot analysis and quantification of DAO (J) and I-FABP (K) serum protein levels in *Atf5*^*flox/flox*^ and *Atf5*^*ΔIEC*^ mice, with or without intraperitoneal CCK injection. Coomassie-stained SDS-PAGE gels are shown as loading controls. Data represent mean ± standard error of the mean (n = 4; *p < 0.05, **p < 0.01, ***p < 0.001 using the Student’s t test). (L) Survival of *Atf5*^*flox/flox*^ and *Atf5*^*ΔIEC*^ mice, with or without intraperitoneal CCK injection, challenged with *Salmonella* (*n = 5*). See Table S1 for all statistics pertaining to survival analysis.

We used a transcriptomic approach to explore how ATF5 regulates leptin availability by analyzing gene expression changes in *Atf5*^*ΔIEC*^ versus control mice small intestine. We found that ATF5 regulates a diverse set of genes in the mammalian intestine with functions in metabolism, immune defense, and signaling, among others (Figures 5A and S5). We conducted upstream regulatory molecule (URM) activity predictions using an ingenuity pathway analysis (Figure 5B) to interpret the functional relevance of differentially expressed genes in *Atf5*^*ΔIEC*^ mice. URM predictions highlighted the dysregulation of key cytokines (including interleukin 10 receptor subunit alpha [*IL10RA*], which is implicated in inflammatory bowel disease^62^), as well as leptin signaling in *Atf5*^*ΔIEC*^ mice. Similarly, analyses of KEGG pathway enrichment of differentially expressed genes in *Atf5*^*ΔIEC*^ mice indicated the dysregulation of pathways linked to inflammatory bowel disease (Figure 5C).

Based on our gene expression data implicating leptin signaling dysregulation, we were particularly intrigued by one differentially expressed gene, cholecystokinin (*Cck*), which was transcriptionally reduced in *Atf5*^*ΔIEC*^ mice. CCK is a gastrointestinal peptide hormone associated with the mammalian satiety response.^63,64^ In addition to being expressed in the small intestine, CCK was previously shown to control circulating leptin levels.^65^ We confirmed that intestinal *Cck* transcript expression is significantly reduced in *Atf5*^*ΔIEC*^ samples (based on quantitative PCR), suggesting that *Cck* may be positively regulated by ATF5 (Figure 5D). We also observed significantly lower CCK protein in *Atf5*^*ΔIEC*^ serum samples compared with control animals (Figure 5E), consistent with our gene expression data. We next performed intraperitoneal injections of CCK peptide to examine whether reduced CCK supply was the cause of the impaired satiety response observed in *Atf5*^*ΔIEC*^ mice. We first measured the effect of CCK administration on circulating leptin levels and found that serum leptin levels of *Atf5*^*ΔIEC*^ mice were restored after injection of CCK peptide (Figure 5F). Furthermore, CCK peptide administration also re-established normal bodyweight and feeding behavior in *Atf5*^*ΔIEC*^ mice (Figures 5G and 5H). Therefore, our data suggest that intestinal ATF5 regulates leptin availability and the satiety response via the transcriptional regulation of *Cck*.

We next determined whether the impaired satiety response resulting from reduced CCK was the cause of intestinal barrier dysfunction seen in *Atf5*^*ΔIEC*^ mice. Indeed, we observed improved intestinal barrier function in *Atf5*^*ΔIEC*^ mice supplemented with CCK, as per the FITC-dextran assay (Figure 5I). Also, the increase in serum biomarkers of intestinal permeability observed in *Atf5*^*ΔIEC*^ mice was suppressed after CCK administration (Figures 5J and 5K). Finally, intraperitoneal CCK injection significantly improved the survival of *Atf5*^*ΔIEC*^ mice challenged with *Salmonella* to that observed with *Atf5*^*flox/flox*^ control animals (Figure 5L). These findings indicate that CCK is a critical mediator of intestinal barrier function regulation by ATF5 through its ability to control leptin availability and the satiety response.

### ATF5 regulates intestinal barrier function by preventing hyperglycemia-associated alterations to glucose metabolism

**Figure 6.**
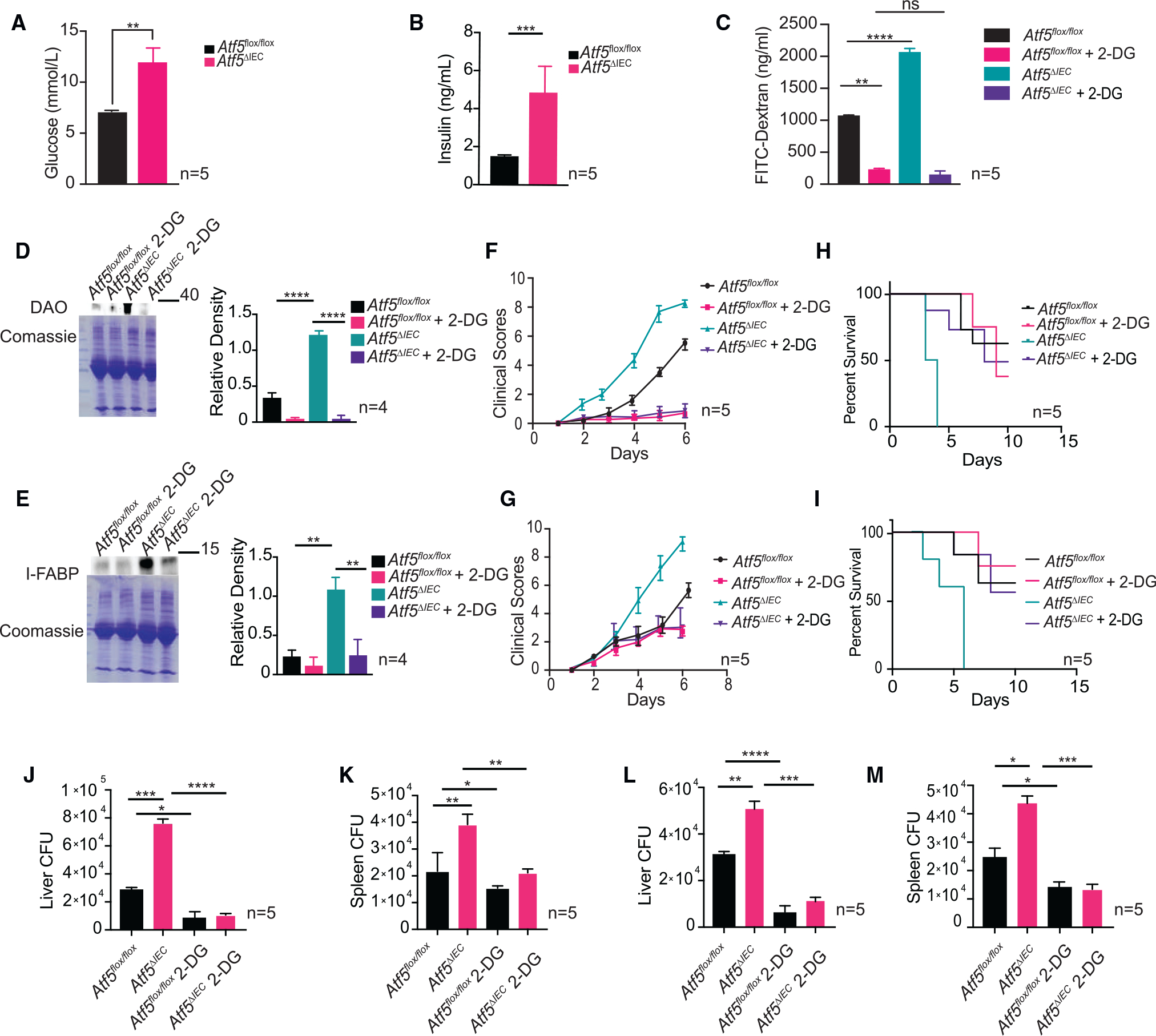
ATF5 supports intestinal barrier function by preventing hyperglycemia and aberrant glycolytic flux (A and B) Blood glucose (A) and insulin (B) levels in *Atf5*^*flox/flox*^ and *Atf5*^*ΔIEC*^ mice. Data represent mean ± standard error of the mean (n = 5; **p < 0.01, ***p < 0.001 using the Student’s t test). (C) Serum FITC-dextran levels in *Atf5*^*flox/flox*^ and *Atf5*^*ΔIEC*^ mice intraperitoneally injected with 2-DG. Data represent mean ± standard error of the mean (n = 5; ns, non-significant using the Student’s t test). (D and E) Immunoblot analysis and quantification of DAO (D) and I-FABP (E) serum protein levels in *Atf5*^*flox/flox*^ and *Atf5*^*ΔIEC*^ mice, with or without intraperitoneal 2-DG injection. Coomassie stained SDS-PAGE gels are shown as loading controls. Data represent mean ± standard error of the mean (n = 4; **p < 0.01, ****p < 0.0001 using the Student’s t test). (F and G) Clinical scores in *Atf5*^*flox/flox*^ and *Atf5*^*ΔIEC*^ mice, with or without intraperitoneal 2-DG injection, challenged with *Salmonella* (F) or *C*. *rodentium* (G). Data represent mean ± standard error of the mean (n = 5; ns, non-significant using the Student’s t test). (H and I) Survival of *Atf5*^*flox/flox*^ and *Atf5*^*ΔIEC*^ mice, with or without intraperitoneal 2-DG injection, challenged with *Salmonella* (H) or *C*. *rodentium* (I) (n = 5). See Table S1 for all statistics pertaining to survival analysis. (J–M) CFU counts of liver and spleen samples from *Salmonella*- (J and K) or *C*. *rodentium*-infected (L and M) *Atf5*^*flox/flox*^ and *Atf5*^*ΔIEC*^ mice, with or without intraperitoneal 2-DG injection. Data represent mean ± standard error of the mean (n = 5; *p < 0.05, **p < 0.01, ***p < 0.001, ****p < 0.0001 using the Student’s t test).

Our data suggest that ATF5 prevents intestinal barrier dysfunction by promoting a CCK/leptin-mediated satiety response. We next explored how the regulation of satiety by ATF5 promoted intestinal barrier integrity. A recent study discovered that obese leptin knockout mice were predisposed to enteric infection because of impaired intestinal barrier regulation.^53^ In this case, the hyperglycemia associated with obesity seemed to be responsible for the impaired barrier function. Specifically, abnormal glucose metabolism resulting from hyperglycemia was found to stimulate a transcriptional reprogramming in intestinal epithelial cells that drove a dysfunctional intestinal barrier.^53^ We hypothesized that a similar mechanism might help to explain the impaired intestinal barrier function of *Atf5*^*ΔIEC*^ mice that predisposes them to enteric infection. We first determined whether *Atf5*^*ΔIEC*^ mice displayed signs of hyperglycemia. As expected, we observed higher serum glucose levels in fed *Atf5*^*ΔIEC*^ mice compared with control animals (Figure 6A). Correspondingly, *Atf5*^*ΔIEC*^ mice also exhibited hyperinsulinemia under fed conditions when compared with control animals (Figure 6B).

Having confirmed the hyperglycemic status of the *Atf5*^*ΔIEC*^ mice, we next asked whether increases in glycolytic flux were responsible for the compromised intestinal barrier of these mutant mice. We used 2-deoxyglucose (2-DG) for this purpose, a synthetic glucose analog that interferes with glycolysis.^66^ Impressively, treatment of *Atf5*^*ΔIEC*^ mice with 2-DG restored normal intestinal barrier function, as reflected by the FITC-dextran assay (Figure 6C), and decreased the presence of serum epithelial permeability biomarkers (Figures 6D and 6E). Based on these findings, we hypothesized that the susceptibility of *Atf5*^*ΔIEC*^ mice to enteric infection was ultimately due to impaired intestinal barrier function resulting from hyperglycemia and uncontrolled glycolytic flux. Indeed, *Atf5*^*ΔIEC*^ mice administered with 2-DG had significantly improved clinical scores after challenge with *Salmonella* or *C*. *rodentium* (Figures 6F and 6G). We also observed a dramatic improvement in the survival of *Atf5*^*ΔIEC*^ mice during enteric infection with these pathogens in the presence of 2-DG (Figures 6H and 6I), coupled with reduced pathogen dissemination to distal organs (Figures 6J–6M).

## DISCUSSION

In the invertebrate model organism *C*. *elegans*, the UPR^mt^ protects the host during pathogen infection by promoting mitochondrial recovery and the induction of innate immunity-related antimicrobial effectors.^16,20,22,23^ Here, we find that the mammalian UPR^mt^ regulator ATF5 functions similarly in protecting the host during enteric pathogen infection, except that it does so by a mechanism involving the maintenance of intestinal barrier function. By transcriptionally controlling the expression of the gut-derived hormone *Cck*, ATF5 is able to positively regulate serum leptin levels, thus stimulating satiety and preventing obesity. The regulation of appetite by ATF5 ultimately promotes intestinal barrier function by preventing alterations to glucose metabolism caused by hyperglycemia. The promotion of intestinal barrier function by ATF5 through the regulation of leptin-mediated satiety response is consistent with previous findings demonstrating a protective role of this hormone pathway in intestinal homeostasis. This includes the promotion of mucosal defenses, colonic epithelial proliferation, and protection against tissue injury.^67–69^

The intestinal epithelium plays an essential role in preventing infiltration of gut luminal contents and enteric pathogens. The ability of the intestinal epithelium to act as a physical barrier is achieved due to the presence of tight junctions and adherens junctions such as ZO-1 and E-cadherin, respectively.^70^ Disruptions to intestinal barrier function are a hallmark of inflammatory bowel diseases (IBD), including Crohn disease and ulcerative colitis.^71^ A number of factors, both genetic and/or environmental, have been associated with decreased intestinal barrier homeostasis and the onset of IBD, including dysfunction to mitochondria.^72^ For example, intestinal epithelia from IBD patients display abnormal mitochondrial morphology before inflammation,^73^ decreased ATP levels,^74,75^ and increased mitochondrial ROS,^76^ indicative of altered mitochondrial function. Mitochondrial function is also required to promote tight junction formation, which is a highly energy-dependent process.^77^ In addition, multiple studies have shown a clear relationship between mitochondrial dysfunction and a perturbed epithelial barrier function. For example, increased mitochondrial oxidative stress has been shown to exacerbate colitis symptoms, which can be alleviate through the administration of mitochondrial-targeted antioxidants.^78^ Heightened oxidative stress because of reduced mitochondrial function also disrupts tight and adherens junction formation.^79,80^ Moreover, deficiencies in multiple regulators of mitochondrial function are known contributors to the intestinal inflammatory process. This includes the master regulator of mitochondrial biogenesis PGCα1^81^ and SLC22A5, which functions as a carnitine transporter involved in fatty acid oxidation.^82^ Last, mitochondrial dysfunction results in increased inflammation through the activation of signaling pathways such as 5’ adenosine monophosphate-activated protein kinase.^83^ The mitigation of mitochondrial dysfunction by ATF5 through the activation of the UPR^mt^ is consistent with its role in supporting intestinal barrier function. However, we believe that the prevention of a hyperglycemic state through the regulation of the satiety response is the predominant mechanism of protection, considering the almost complete rescue of *Atf5*^*ΔIEC*^-associated intestinal barrier dysfunction via supplementation with satiety hormones or through administration of 2-DG.

The UPR^mt^ has been previously associated with the regulation of intestinal inflammation, whereby Rath et al.^84^ suggested that the kinase double-stranded-RNA-activated protein kinase (PKR) positively regulates the UPR^mt^ in a DSS model of colitis. In this study, mice that were deficient for PKR were more resistant to DSS-induced colitis.^84^ PKR represents one of four kinases of the integrative stress response that decreased global translation rates via phosphorylation of the translation initiation factor eIF2α during stress.^85^ A decrease in global translation results in the preferential translation of specific mRNAs that contain upstream open reading frames (uORFs).^86^ ATF5 is among these mRNAs that harbor an uORF and whose translation is increased during stress.^87^ ATF5 is also transcriptionally upregulated by ATF4 and CHOP,^88^ both of which harbor uORFs that are preferentially translated during mitochondrial stress.^89^ Our current study of the UPR^mt^ regulator ATF5, therefore, presents seemingly contradictory findings, particularly that the UPR^mt^ and ATF5 are required for protection against DSS-associated intestinal barrier disruption. However, in contrsast with the findings of Rath et al.,^84^ a subsequent study concluded that PKR protected animals from DSS-associated colitis.^90^ Furthermore, the upregulation of ATF5 is only one of many downstream effectors of PKR.^91^ It is, therefore, possible that these effectors have varied impacts that may explain the contrasting findings of PKR relative to ATF5. Future investigations that examine the contributions of each regulatory effector downstream of PKR signaling would be valuable for resolving the roles of PKR in the context of intestinal barrier maintenance.

The relationship between altered glucose metabolism and decreased intestinal barrier function also remains unclear and warrants further study. A previous transcriptomic analysis of hyperglycemic mice demonstrated a clear reduction in the expression of genes associated with protein N-glycosylation. It was proposed that reduced N-glycosylation could impact multiple epithelial functions that may be important in the establishment of the intestinal barrier.^53^ Our transcriptomic analyses identified many genes that show significant differential expression with the loss of ATF5. Among the genes that are downregulated in *Atf5*^*ΔIEC*^ mice include those that encode enzymes mediating various steps in protein glycosylation. The downregulation of these genes is consistent with the hyperglycemic state of *Atf5*^*ΔIEC*^ mice and might account for the decreased intestinal barrier function of these mutant animals.

In conclusion, our study significantly expands our understanding of the physiological roles and functional interactions mediated by ATF5 and the UPR^mt^ in mammals. We show that ATF5 possesses a conserved role in protecting the host during infection similar to ATFS-1 in *C*. *elegans*. In mammals, intestinal ATF5 and the UPR^mt^ protect the host by maintaining the integrity of the intestinal epithelium, thus establishing a barrier that prevents the infiltration of microbes and toxins. This is achieved metabolically by preventing aberrant glucose metabolism through control of the satiety response. Whether ATF5 and the UPR^mt^ possess additional functions in other cell types to protect the host during infection would be valuable to explore in future studies.

### Limitations of the study

While we show that ATF5 promotes intestinal barrier function by regulating the satiety response and preventing hyperglycemia, we are still unclear of the exact relationship at a mechanistic level. We posit that the hyperglycemia results in reduced transcript abundance of genes involved in protein glycosylation, as discovered previously.^53^ However, the aforementioned study, for example, discovered that all genes relating to the N-glycosylation pathway were downregulated in the hyperglycemic state.^53^ In contrast, our transcriptomic analysis of *Atf5*^*ΔIEC*^ intestinal tissue only identified a handful of genes with glycosylation properties. It remains possible that our transcriptomic analysis did not have the proper resolution to detect differences in abundance that might have been mild. A biochemical analysis of the glycosylation state of key intestinal epithelium junctional proteins in the presence or absence of ATF5 is warranted to validate this putative model.

Our study also did not investigate for changes in the intestinal microbiome that might occur in the absence of ATF5, which could potentially impact the integrity of the intestinal barrier. There is established cross-talk between the host microbiome and mitochondria, including how mitochondria can influence gut microbiome diversity via production of ROS.^92^ We observed in our current study that ATF5 promotes mitochondrial recovery in the intestinal epithelium, including a role in limiting oxidative damage. Presumably, then, ROS production is altered in the absence of ATF5, which may impact the host gut microbiome and possibly intestinal barrier function. Further work is needed to characterize gut microbiome diversity in the presence or absence of ATF5, and how this might influence intestinal barrier maintenance.

## STAR★METHODS

### RESOURCE AVAILABILITY

#### Lead contact

Further information and requests for resources and reagents should be directed to and will be fulfilled by the Lead Contact, Mark W. Pellegrino (mark.pellegrino@uta.edu).

#### Materials availability

All unique/stable reagents generated in this study are available from the lead contact with a completed Materials Transfer Agreement.

#### Data and code availability

- All data supporting the findings from this study are available in the main manuscript or supporting files. The RNA-seq raw data are available from the NCBI SRA database with the accession details provided in key resources table.
- This paper does not report original code.
- Any additional information required to reanalyze the data reported in this paper is available from the lead contact upon request.

### EXPERIMENTAL MODEL AND SUBJECT DETAILS

#### Animal models

All animal studies were carried out following the recommendations in the Guide for the Care and Use of Laboratory Animals, 8th Edition (National Research Council), and were approved by the University of Texas at Arlington Animal Care and Use Committee. Animals were euthanized either before the onset of clinical disease or at the initially defined humane endpoint (clinical signs including ruffled coat, hunched posture, and weight loss).

Mice were fed a chow diet (2018 Teklad Diet) and were housed with a 12-h:12-h light-dark cycle with *ad libitum* access to food and water. Mice were housed with sterile bedding in sterile microisolator cages. Experiments were performed with age- (2–4 months) and sex-matched (males) mice, unless otherwise specified. *Atf5*^*flox/flox*^ mice, which contain *loxP* sites flanking exon 3 of the *Atf5* gene, were crossed with the *B6.Cg-Tg(Vil1-cre)1000Gum/J* mouse (Villin^Cre^) (The Jackson Laboratory; Bar Harbor, ME) that express the Cre recombinase gene under the control of the *villin* promoter, generating *Atf5*^*ΔIEC*^ mice (Cyagen Inc.). The presence of the *Atf5* deletion was confirmed by PCR genotyping of tail clips at 21 days using primers Atf5-KO.1s (GCAGGATTACAGACGTGGGAGCAG) and Atf5-KO.2AS (AGGTCTTCACTGAAAGCGGTATGC), in addition to verifying the presence of Cre recombinase using primers Region-Cre.1s (CATATTGGCAGAACGAAAACGC) and Region-Cre.2AS (CCTGTTTCACTATCCAGGTTACGG). *Atf5*^*flox/flox*^ mice were used as controls for all experiments. Mice body weight measurements began at six weeks of age and were followed over a month period. Animals had ad libitum access to rodent chow. Weight and food intake were determined daily.

#### Bacterial strains

*Salmonella enterica* subsp. *enterica* (serovar Typhimurium) (ATCC 700720) and *Citrobacter rodentium* (ATCC 51459) were used for infection studies. Bacteria were diluted in sterile PBS to achieve the correct inoculum of (10^6^–10^9^). *C. rodentium* was grown overnight in LB media at 37°C. Cultures were centrifuged and the resulting pellet resuspended in sterile PBS to the desired dose.

### METHOD DETAILS

#### Western blot analysis

For Western blot analysis, 15 μg protein samples were run on 12% SDS-PAGE gels, wet-transferred to nitrocellulose, and visualized with Ponceau S. Blocking was performed with 5% milk and antibodies were incubated in 5% milk in PBS-T. Images were acquired using an Azure Biosystems C200 apparatus and analyzed in ImageJ. The following antibodies were used at a dilution of 1:1000: ATF5 (AbCam), actin (Cell Signaling), HSP60 (Cell Signaling), AFG3L2 (Cell Signaling), LonP (Cell Signaling), E-cadherin (Cell Signaling), ZO-1 (Proteintech), DAO (Invitrogen), I-FABP (Invitrogen), Lrg5 (Invitrogen), Sox9 (Cell Signaling).

#### Mitochondrial function assays

##### Oxygen consumption rate (OCR) assay

The OCR assay was performed using the MitoXpress Xtra oxygen consumption assay kit (Agilent, USA) and isolated intestinal tissue that was harvested and flushed with PBS. Equally-weighted tissue samples were transferred to wells of a 96-well plate in a final sample volume of 90 μL of warmed DMEM. The oxygen probe was then added to each sample at a volume of 10 μL. The 96-well plates were then sealed with two drops of mineral oil and sample wells were immediately read on a Synergy *Neo* 2 plate reader using Gen5 software (BioTek, Wisnooski, VT, USA) in a time-resolved fluorescence mode with 380 nm excitation and 650 nm emission filters. Data were collected for 1 h at 25°C. To eliminate background oxygen consumption, controls (in triplicates) with mitochondrial experimental buffer and mitochondrial experimental buffer plus oxygen probe with no sample, were included in each respiration assay. The measured time profiles of fluorescence from each sample were normalized to the signal at time zero to obtain normalized intensity. The slope of each sample, which reflects mitochondrial OCR, was determined by selecting the linear portion of the signal profile and applying the linear regression according to the probe’s manufacturer’s instructions. Calculated slopes were used to determine the respiration rates in each sample. The relative respiration rates were calculated as follows: R = (Ss−Sn)/Sp, where Ss, Sn and Sp is the slope of the sample, the negative control and the positive control, respectively. Each condition was analyzed in three replicates on the 96-well plates. The OCR experiment was repeated three times.

#### Quantification of mitochondrial membrane potential

TMRE-Mitochondrial membrane potential assay kit (Abcam) was used to measure membrane potential. Cells were first incubated with culture medium containing 200 nM TMRE for 30 min at 37°C, rinsed three times with PBS and 0.2% BSA, and incubated with culture medium containing 50 nM TMRE. TMRE fluorescence was measured using a Synergy HTX microplate reader (BioTek) following the manufacturer’s instruction.

#### ATP production quantification

ATP was quantified using a bioluminescence ATP measurement kit (Thermo Fisher Scientific). Mice intestinal samples were heated at 95°C for 15 min, then placed in ice for 5 min. Samples were then centrifuged at 14,000 × *g* for 10 min at 4°C and the supernatant recovered for ATP measurement. 10 μL of each sample in duplicate were transferred to 96-well plates. 90 μL of ATP assay solution (prepared according to the manufacturer’s instructions) were added to each sample. Sample wells were then read on a Synergy *Neo* 2 plate reader using Gen5 software (BioTek, Wisnooski, VT, USA) with a luminometer filter. An ATP standard curve was generated and the ATP concentration for each sample was calculated based on the standard curve.

#### Measurement of oxidative damage

The OxyBlot protein oxidation detection kit (Millipore-Sigma) was used to measure the level of protein oxidation. The DNP reaction mixture was prepared by adding 15 μg intestinal protein sample (7 μL), 3 μL of 15% SDS, and 10 μL of DNP solution. The mixture was kept at room temperature for 15 min, followed by addition of 7.5 μL of neutralization buffer. Samples were then separated on a 10% SDS-PAGE gel, transferred to nitrocellulose (Bio-Rad) and blocked with 5% non-fat milk for 1 h. After washing with 1X PBS, the membrane was incubated with primary antibody (1:150) overnight at 4°C and then secondary antibody (1:300) for 1h at room temperature. Membranes were incubated with ECL plus detection reagent (Bio-Rad) and scanned using a chemiluminescent scanner (Bio-Rad). Band densities in a given lane were analyzed using ImageJ and summated. Afterward, the membranes were incubated with 15% hydrogen peroxide for 30 min at room temperature and treated with anti-actin antibody. OxyBlot values were then normalized to actin for each sample.

#### Immunofluorescence

To prepare samples for immunofluorescence, tissue sections were deparaffinized and hydrated through xylenes and graded alcohol series. Slides were washed with PBS for 5 min and incubated with 2.5% normal goat serum for 20 min. Samples were incubated with anti-ATF5 antibody (1:1000; Abcam) overnight at 4°C. Samples were then washed two times with PBS, one time with PBST, each for 5 min. Slides were then incubated with secondary antibody (1:1000) for 50 min at room temperature, followed by PBS/PBST washing as before. Samples were then incubated with DAPI (1:500) for 10 min, washed three times with PBS for 5 min each, and treated with Sudan black block background for 5 min. Slides were washed under running water for 20 min prior to mounting.

For TUNEL staining, tissue sections were dewaxed, rehydrated, and incubated with proteinase K for 20 min at 37°C. Slides were washed two times with PBS, and then one time with PBST, 5 min each wash. Slides were then treated with 0.1% Triton-PBS for 15 min at room temperature, washed as before, and then incubated with TUNEL reagent (*In situ* cell death detection kit; Roche) for 2 h at 37°C. Slides were washed as before and incubated with DAPI (1:500) for 10 min at room temperature. Slides were washed three times for 5 min each with PBS and mounted for analysis. For BrdU assay, BrdU was diluted to 0.8 mg/mL in sterile drinking water. After 24 h the mice were sacrificed and the tissue fixed and processed. Quantification of TUNEL and BrdU positive cells were based on an automatic threshold analysis of immunofluorescence images to automatically identify the top brightest structures using ImageJ software.

#### CFU measurement

To quantify bacterial loads in the spleen, liver, and intestines, samples of each organ were homogenized, serially diluted in sterile PBS, and seeded onto LB, MacConkey, or *Salmonella-Shigella* agar.

#### Mouse infection/survival analysis

Food was withheld for 4 h prior to infection studies. Mice were treated with streptomycin 24 h before infection using a blunt end straight size 20-gauge gavage needle with 100 μL PBS containing 200 mg/mL streptomycin. For *Salmonella* infection, mice were gavaged with bacteria at a dose of 10^7^ CFU’s; for *C. rodentium,* mice were gavaged at a dose of 10^5^ CFU’s.

For survival analyses, mice were orally inoculated with *Salmonella* at a dose of 10^9^ CFU’s or *C. rodentium* at a dose of 10^8^ CFU’s. The infectious dose was verified by plating serial dilutions on selective MacConkey plates/SS Agar. For survival analysis, the mice were monitored daily and sacrificed if they met any of the following clinical endpoints: 15% body weight loss, hunching, inactivity, ruffled fur or any severe sign of infection.

#### H&E staining and histopathology analyses

Histopathological damage was assessed using various regions of the mouse gastrointestinal system. Tissue portions were fixed in 10% formalin, embedded in paraffin, cut into 3–4 μm thick sections, mounted on glass slides, and finally stained with hematoxylin (Harris hematoxylin) and eosin Y solution (0.5% aqueous).

Histopathologic scores for inflammation were based on the extent of inflammatory cells (above normal populations) within the colon which correlated with the severity of inflammation. Scores ranged from 0 to 4 depending on the predominant location of inflammatory cells (0 = normal populations, 1 = increased cells in the lamina propria, 2 = inflammatory cells in the submucosa, 3 = inflammatory cells in the muscularis, 4 = transmural inflammatory cells).

Scores for edema were based on the predominant location of the edema present within the entire section and ranged from 0 to 3 (0 = no edema present, 1 = edema present in the lamina propria, 2 = edema present in the submucosa, 3 = edema present in the muscularis).

Histologic scores for erosion/ulceration were based on the extent of epithelial damage and ranged from 0 to 3 (0 = no epithelial damage, 1 = only surface epithelium missing, 2 = majority of epithelium damaged with occasional crypts present, 3 = no epithelium remains).

The extent of inflammation, edema, and erosion/ulceration were estimated based on the precent of the sample affected. Scores for extent ranged from 0 to 4 (0 = 0%, 1 = <5% affected, 2 = 5%–25% affected, 3 = 25%–50% affected, 4 = >50% affected.

Epithelial hyperplasia was limited to crypt epithelium and scores were based on the increased thickness of the crypts. Scores ranged from 0 to 3 (0 = no hyperplasia present, 1 = minimal/focal, 2 = mild/regional, 3 = moderate/extensive).

Total scores were calculated for each sample and determined by multiplying the individual scores by their respective extent score and adding all scores together.

#### FITC-dextran permeability assay

Intestinal permeability was assessed by oral administration of FITC-dextran (Sigma). Food and water were withdrawn for 4 h prior to study onset. Mice were subsequently gavage-fed the FITC-dextran solution at 80 mg/100 g bodyweight. Serum was collected 5 h post-gavage feeding, and FITC-dextran fluorescence measurements performed in duplicate on a Synergy *Neo* 2 plate reader using Gen5 software (BioTek). Serial dilutions of FITC-dextran in PBS were used to calculate a standard curve.

#### DSS-induced colitis protocol

Mice were treated with a 4% DSS solution for 7 days via their drinking water, which was changed every 2–3 days. Control mice received water *ad libitum*. Animals were inspected daily for changes in bodyweight, food/fluid consumption, and occurrence of diarrhea and bleeding. The sum of the scores for diarrhea, bleeding, and bodyweight loss was used to calculate the Disease Activity Index (DAI).^93^ Where appropriate, mice were administered doxycycline hyclate in their drinking water (1.5 g/L) for 1 week *ad libitum*, with fresh suspension every 3–4 days. Animals were given only sterile drinking water for a period of 24 h prior to DSS treatment, allowing for efficient clearance of doxycycline from the system.^94^

#### RNA-seq analysis

Total RNA was extracted and purified from whole small intestine using SV Total RNA Isolation Extraction system (Promega). RNA integrity was assessed using the Bioanalyzer 2100 system. RNA-seq libraries were generated by Novogene Inc. (CA, USA) using the NEBNext Ultra RNA Library Kit from Illumina (NEB) following the manufacturer’s protocol. RNA-seq libraries were sequenced on an Illumina Hiseq 4000 using paired-end 150-bp reads. Raw reads were processed by removing reads containing Ilumina adapters, reads containing poly-Ns, and low-quality reads (<Q30) using the program Trimmomatic.^95^ Processed reads were aligned to the *M. musculus* reference genome using TopHat v.2.0.9.^96^ Mapped reads from each sample were used to estimate the number of reads mapped to each gene, which were then converted to reads per kilobase million (RPKM) using Cufflinks v.2.1.1. and HTSeq v.0.6.1. Pairwise differential expression analysis was performed using *DESeq*2 R package (v.1.10.1).^97^ Statistical significance for differentially expressed genes was calculated using Benjamini-Hochberg corrected p values (*Padj*), with a threshold of <0.05 considered as significant. Heatmaps were generated with the pheatmap R package (v1.0.12). Upstream Regulatory Molecule analysis was performed using Ingenuity Pathway Analysis (IPA) (Qiagen Inc.), and KEGG and GO term analyses were performed with the clusterProfiler (v4.2.2) and enrichplot (v1.14.2) R packages.^98,99^

#### Quantitative PCR

cDNA was generated using the iScript cDNA synthesis kit (Bio-Rad, Hercules, CA). SsoAdvanced Universal SYBR Green Supermix (Bio-Rad) was used for qPCR reactions and measured on a CFX connect real-time PCR system (Bio-Rad). *Cck* qPCR was performed using primers Cck-qPCR.3s (GGAGCTCACGAACCCAATTT) and Cck-qPCR.4AS (CATGTAGTCCCGGTCACTTATTC). Quantification cycle (Cq) of each RT-qPCR target was normalized to the reference gene *Hprt* using primers Hprt1-qPCR.1s (TGACACTGGTAAAACAATGCA) and Hprt1-qPCR.2AS (GGTCCTTTTCACCAGCAAGCT). Data are presented in *Atf5*^*flox/flox*^ vs *Atf5*^*−ΔIEC*^, as relative Cq (i.e., Cq).

#### Measurement and administration of leptin and CCK

Six week old mice were sacrificed and plasma leptin and cholecystokinin were measured using the Quantikine ELISA mouse Leptin Immunoassay kit (R&D Systems) and the Quantikine ELISA mouse Cholecystokinin ELISA Immunoassay kit (R&D systems), respectively, according to the manufacturer’s protocol. A microplate scanning spectrometer (BioTek) was used to measure the optical density for leptin and CCK (450 nm wavelength correction: 540 nm–570 nm).

Purified leptin (R&D Systems) and CCK (Bachem) were administered to six week old mice intraperitoneally at doses of 5 mg/kg and 10 ug/kg, respectively.

#### Measurement of glucose/insulin and administration of 2-DG

Six week old mice were sacrificed and glucose was measured by Contour next One Blood Glucose Monitoring System. Insulin levels were measured using the mouse insulin (INS) ELISA kit (R&D Systems) according to the manufacturer’s instructions. 2-DG (Acros Organics) was administered to mice intraperitoneally at a dose of 5 mg/kg.

### QUANTIFICATION AND STATISTICAL ANALYSIS

Statistical analysis was performed using Graphpad Prism v9. Details on statistical tests are referred to in each figure legend. We considered a comparison statistically different when p values were below 0.05.

## Supplementary Material

1

2

3

## Figures and Tables

**KEY RESOURCES TABLE:** 

REAGENT or RESOURCE	SOURCE	IDENTIFIER

Antibodies		
Recombinant Anti-ATF5	Abcam	Cat# ab184923; RRID:AB_2800462
Anti-beta Actin antibody	Abcam	Cat# Ab8226; RRID:AB_306371
Anti-Hsp60 antibody	Abcam	Cat# ab46798; RRID:AB_881444
AFG3L2 Polyclonal	Proteintech	Cat# 14631-1-AP; RRID:AB_2242420
LONP1 Polyclonal	Proteintech	Cat# 15440-1-AP; RRID:AB_2137152
E-Cadherin Rabbit mAb	Cell Signaling	Cat# 3195; RRID:AB_2291471
Anti-ZO1 tight junction protein	Abcam	Cat# ab216880; RRID:AB_2909434
Recombinant Anti-I-FABP	Abcam	Cat# ab128860; RRID:AB_11140210
DAO Polyclonal	ThermoFisher Scientific	Cat# PA5-58482; RRID:AB_2640377
Recombinant Anti-Ki67	Abcam	Cat# ab16667; RRID:AB_302459
Recombinant Anti-LGR5	Abcam	Cat# ab75850; RRID:AB_1523716
Sox9 Rabbit mAb	Cell Signaling	Cat# 82630; RRID:AB_2665492

Bacterial and virus strains		

*Salmonella enterica* subsp. *enterica* (serovar Typhimurium)	ATCC	700720
*Citrobacter rodentium*	ATCC	51459

Chemicals, peptides, and recombinant proteins		

Rm Leptin	R & D Systems	Cat. # 498-OB
2-Deoxy-D-glucose	Sigma	Cat. # D6134-5G
Cholecystokinin	Bachem	Cat. # H-2080.0001
5-Bromo-2′-deoxyuridine	ThermoFisher Scientific	Cat. # 228590025

Critical commercial assays		

MitoXpress Xtra Oxygen Consumption Assay	Agilent	Cat.# MX-200-4
SV Total RNA Isolation Kit	Promega	Cat.# Z3100
Rat/Mouse Insulin ELISA Kit	Millipore	Cat. # EZRMI-13K
Quantikine ELISA Mouse/Rat Leptin	R & D Systems	Cat. # MOBooB
Oxyblot Protein Oxidation Detection Kit	Millipore	Cat. # S7150

Deposited data		

Raw RNA sequencing data	NCBI SRA database	Database: SAMN26514638

Experimental models: Organisms/strains		

*Atf5^flox/flox^*	Cyagen Inc.	This manuscript
B6.Cg-Tg(Vil1-cre)1000Gum/J	The Jackson Laboratory	Strain #021504

Oligonucleotides		

Atf5-KO.1s: GCAGGATTACAGACGTGGGAGCAG	IDT	This manuscript
Atf5-KO.2AS: AGGTCTTCACTGAAAGCGGTATGC	IDT	This manuscript
Region-Cre.1s: CATATTGGCAGAACGAAAACGC	IDT	This manuscript
Region-Cre.2AS: CCTGTTTCACTATCCAGGTTACGG	IDT	This manuscript
Cck-qPCR.3s: GGAGCTCACGAACCCAATTT	IDT	This manuscript
Cck-qPCR.4AS: CATGTAGTCCCGGTCACTTATTC	IDT	This manuscript
Hprt1-qPCR.1s: TGACACTGGTAAAACAATGCA	IDT	This manuscript
Hprt1-qPCR.2AS: GGTCCTTTTCACCAGCAAGCT	IDT	This manuscript

Software and algorithms		

Graphpad Prism 9	Graphpad	https://www.graphpad.com/scientific-software/prism/
ImageJ	ImajeJ	https://imagej.nih.gov/ij/
Biorender	Biorender	https://biorender.com/
pheatmap	pheatmap	https://cran.r-project.org/web/packages/pheatmap/index.html
Ingenuity Pathway Analysis (IPA)	Qiagen Inc.	https://digitalinsights.qiagen.com/products-overview/discovery-insights-portfolio/analysis-and-visualization/qiagen-ipa/
clusterProfiler	Wu et al., 2021	https://bioconductor.org/packages/clusterProfiler/
Enrichplot	Yu, 2012	https://yulab-smu.top/biomedical-knowledge-mining-book/

Other		

Contour Next One Blood Glucose Monitoring System	Ascensia Diabetes Care	Cat.# 9763- 7955930

## Highlights

- ATF5 protects against enteric infection as part of the mitochondrial UPR
- ATF5 promotes intestinal barrier function to defend against enteric infection
- ATF5 fosters the satiety response through regulation of cholecystokinin (*Cck*)
- ATF5-mediated satiety averts hyperglycemia-induced intestinal barrier dysfunction
