# Supplementary material for: The mitochondrial UPR regulator ATF5 promotes intestinal barrier function via control of the satiety response
Source: Cell Rep. Author manuscript; Available in PMC 2023 Jan 1. (PMC9805788; doi:10.1016/j.celrep.2022.111789)
Supplement: 1 [file NIHMS1857901-supplement-1.pdf]

Cell Reports, Volume *41*

Supplemental information

The mitochondrial UPR regulator ATF5 promotes intestinal barrier function via control of the satiety response

Douja Chamseddine, Siraje A. Mahmud, Aundrea K. Westfall, Todd A. Castoe, Rance E. Berg, and Mark W. Pellegrino

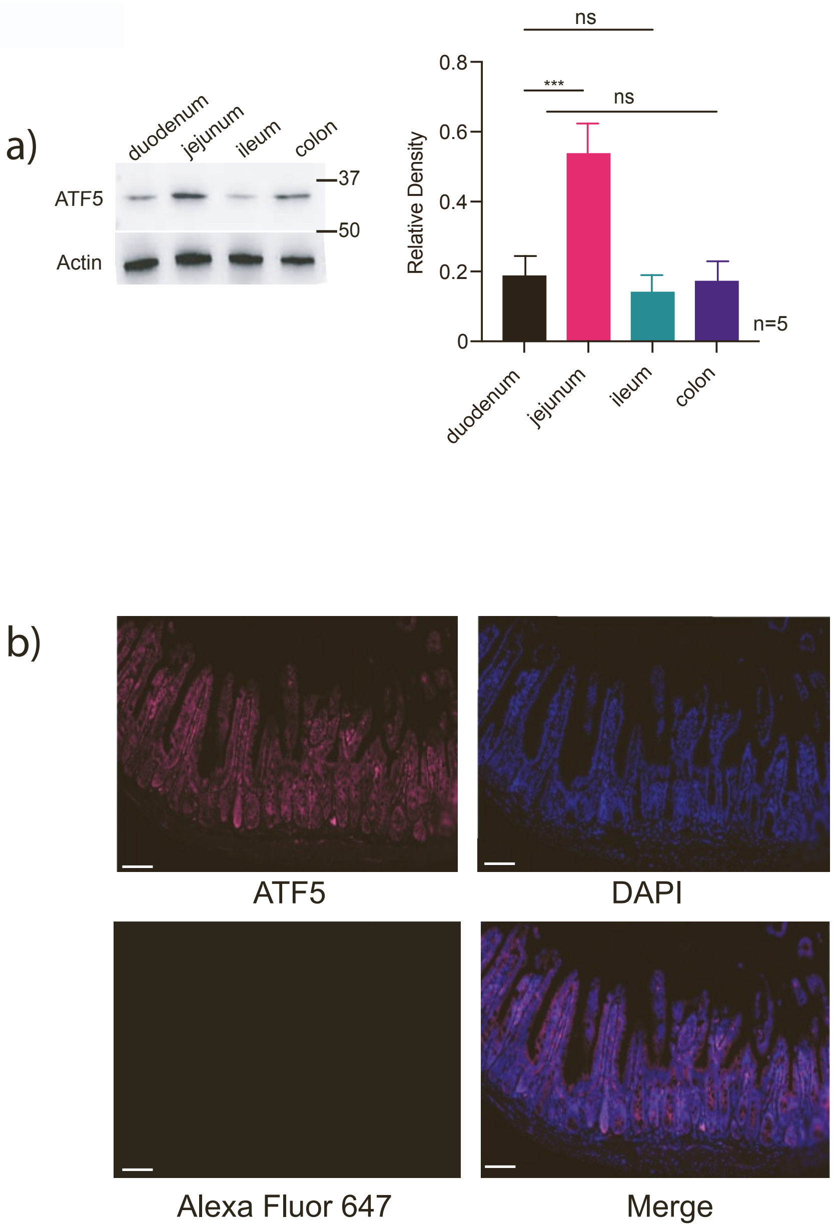

Figure S1. ATF5 is expressed in the mammalian intestine.

a. Immunoblot analysis and quantification of ATF5 in various intestinal segments. Data represent mean $\pm$ SEM (*n=5; ns,* non-significant, *** denotes *p<0.001,* using Student's t-test). Scale bar indicates 100 µm.

b. Immunohistochemistry of small intestine tissue sections from *Atf5*$^{flox/flox}$ mice. Tissue sections were co-stained with anti-ATF5 antibody and DAPI. A secondary antibody-only control is also included (*n=3*). Scale bars denote 100 µm.

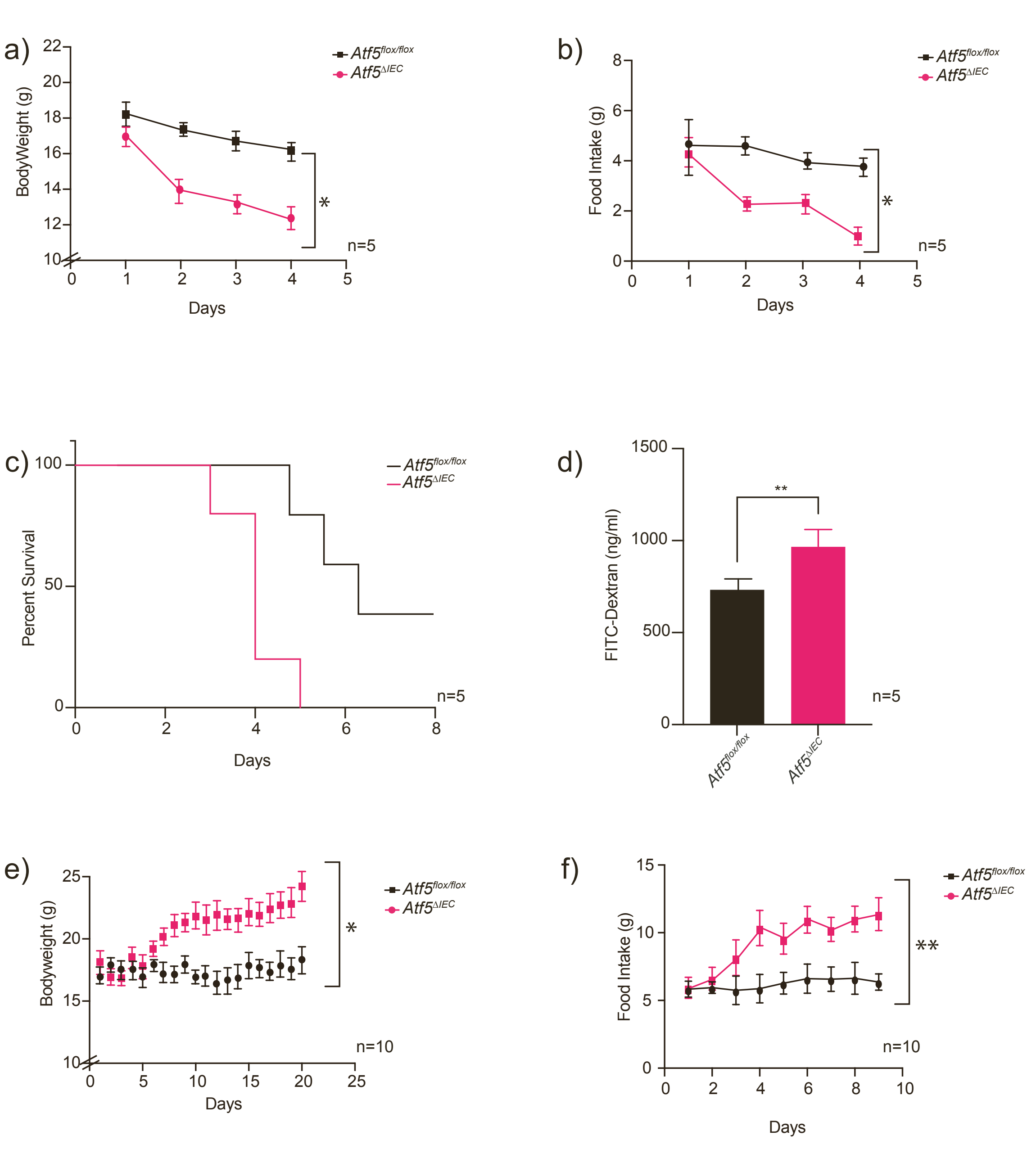

Figure S2. ATF5 protects against enteric infection and regulates bodyweight irrespective of sex.

a, b. Changes in bodyweight (a) and feeding (b) of *Atf5*$^{flox/flox}$ and *Atf5*$^{\Delta IEC}$ female mice during challenge with *Salmonella*. Data represent mean $\pm$ SEM (*n=5;* * denotes *p*<0.05 using Student's t-test).

c. Survival of *Atf5*$^{flox/flox}$ and *Atf5*$^{\Delta IEC}$ female mice during challenge with *Salmonella* (n=5). See Table S1 for all statistics pertaining to survival analysis.

d. Serum FITC-dextran levels in female *Atf5*$^{flox/flox}$ and *Atf5*$^{\Delta IEC}$ mice in the absence of infection. Data represent mean $\pm$ SEM (n=5; ** denotes p<0.01 Student's t-test).

e, f. Changes in bodyweight and feeding in *Atf5*$^{\Delta IEC}$ female mice fed a standard diet (n=10; * denotes p<0.05, ** denotes p<0.01 Student's t-test).

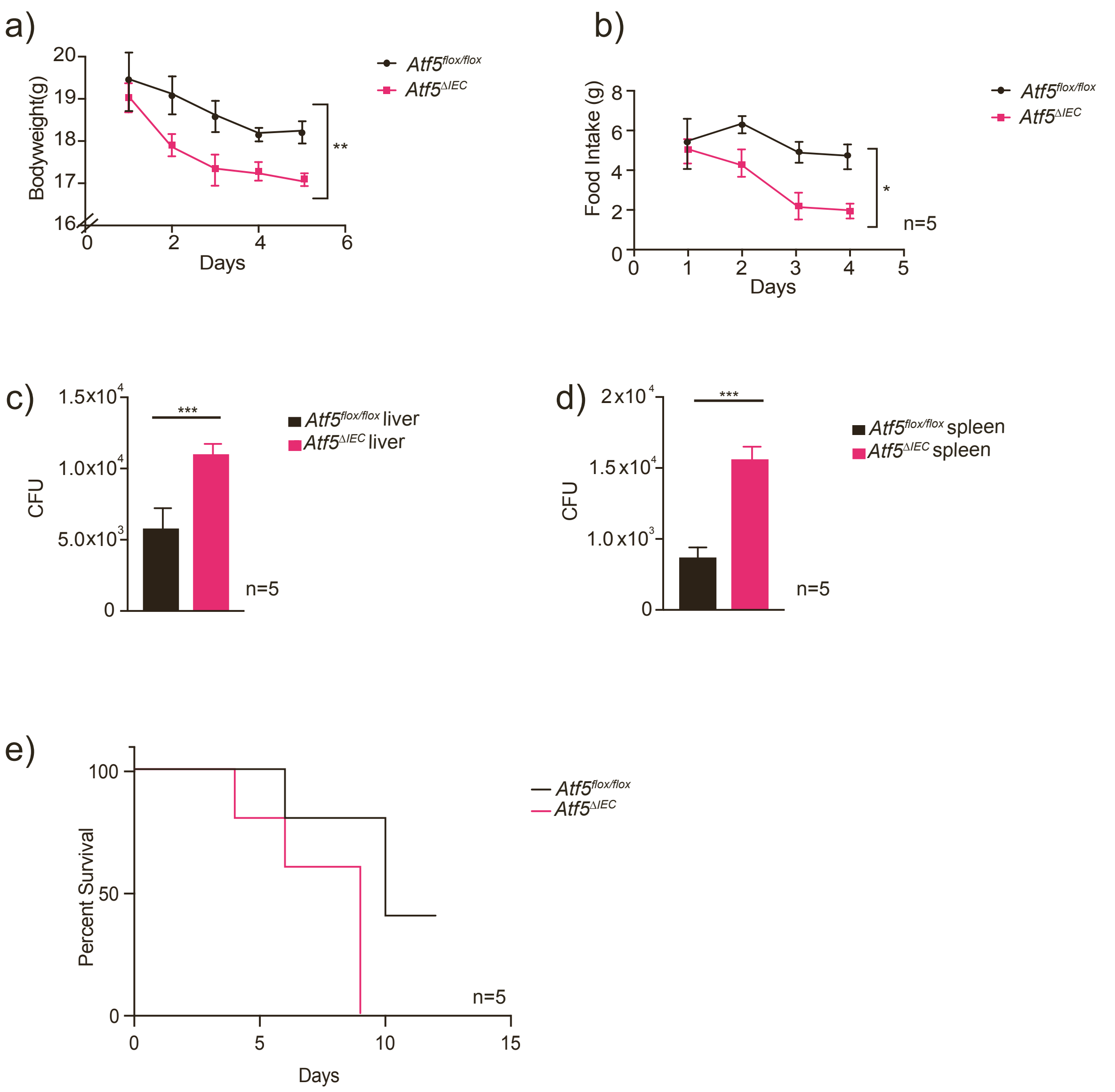

Figure S3. ATF5 protects against enteric infection with *C. rodentium*.

a, b. Changes in bodyweight (a) and feeding (b) of $Atf5^{flox/flox}$ and $Atf5^{\Delta IEC}$ mice during challenge with *C. rodentium*. Data represent mean $\pm$ SEM ($n=5$; * denotes $p<0.05$, ** denotes $p<0.01$ using Student's t-test).

c, d. Colony-forming units (CFU) of liver (c) and spleen (d) samples from *C. rodentium* infected *Atf5*$^{flox/flox}$ and *Atf5*$^{\Delta IEC}$ mice (*n=5;* *** denotes $p<0.001$ using Student's t-test).

e. Survival of *Atf5*$^{flox/flox}$ and *Atf5*$^{\Delta IEC}$ mice during challenge with *C. rodentium* (*n*=5). See Table S1 for all statistics pertaining to survival analysis.

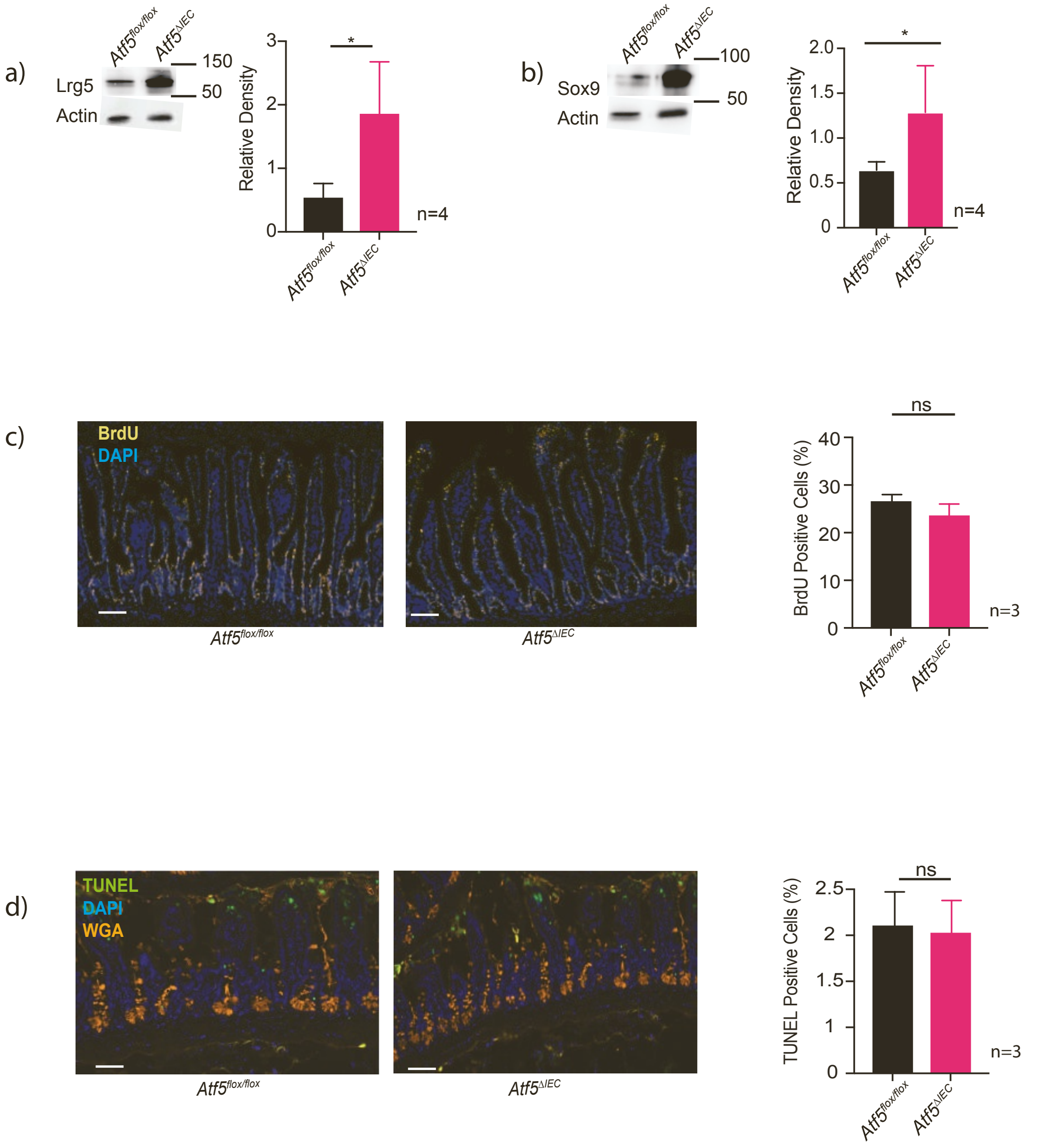

Figure S4. ATF5 does not regulate intestinal cell differentiation, proliferation, or apoptosis.

a, b. Immunoblot analysis and quantification of Lgr5 (a) and Sox9 (b) protein levels in *Atf5*$^{flox/flox}$ and *Atf5*$^{\Delta IEC}$ mice. Actin was used as a loading control. Data represent mean $\pm$ SEM (*n=4;* * denotes *p*<0.05 using Student's t-test).

c, d. Representative images of BrdU- (c) or TUNEL-stained (d) intestinal samples from *Atf5*$^{flox/flox}$ and *Atf5*$^{\Delta IEC}$ mice. Wheat germ agglutinin (WGA) Alexa Flour 488 was used to outline tissue architecture (*n=3; ns,* denotes non-significant using Student's t-test). Scale bars denote 100 μm.

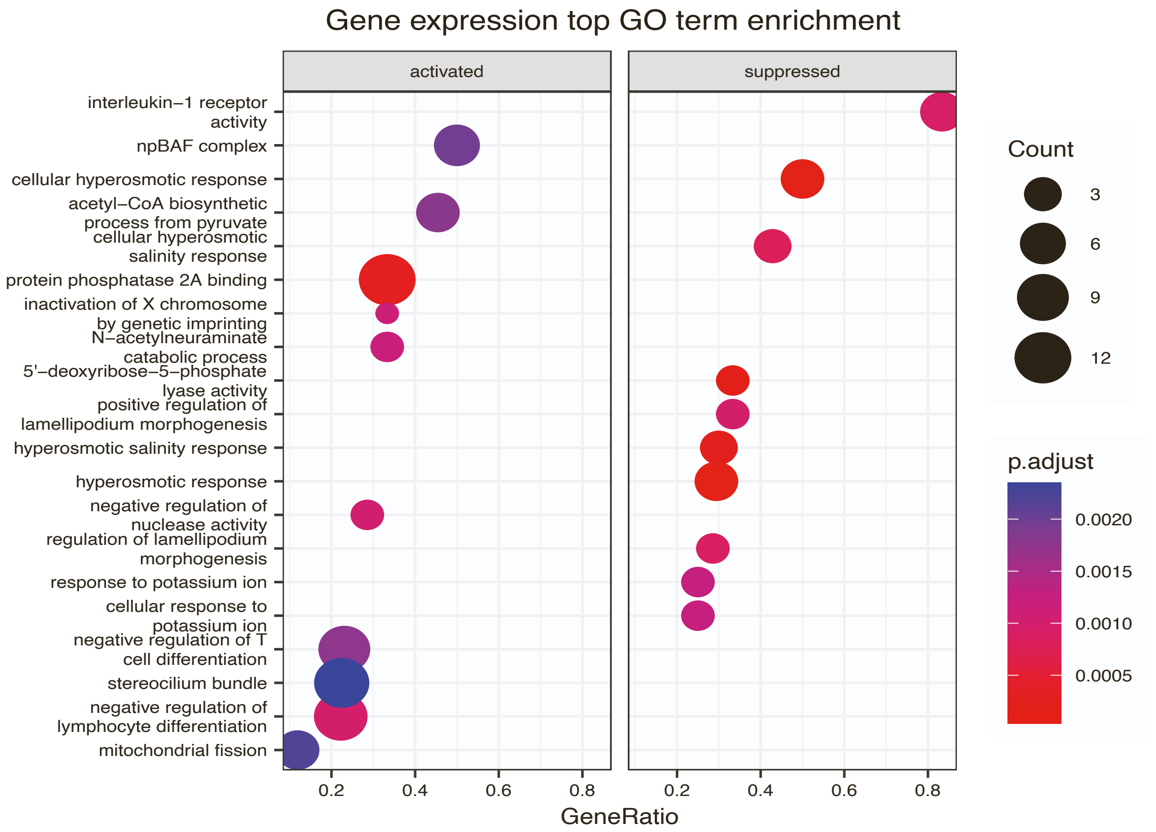

Figure S5. Gene ontology (GO) terms for genes differentially expressed between *Atf5*$^{flox/flox}$ and *Atf5*$^{\Delta IEC}$ mice.

Table S1. Statistical analysis relating to survival data.

Figure	Replicate	Comparison	p-value	value summary
1L	1	ATF5 flox/flox versus ATF5 ΔIEC [Salmonella Typhimirium]	0.007	**
	2	ATF5 flox/flox versus ATF5 ΔIEC [Salmonella Typhimirium]	0.05	*
	3	ATF5 flox/flox versus ATF5 ΔIEC [Salmonella Typhimirium]	0.05	*
3G	1	ATF5 flox/flox versus ATF5 flox/flox [DSS,DSS + Doxy]	0.027	*
	2	ATF5 flox/flox versus ATF5 flox/flox [DSS,DSS + Doxy]	0.027	*
	3	ATF5 flox/flox versus ATF5 flox/flox [DSS,DSS + Doxy]	0.034	*
3H	1	ATF5 flox/flox versus ATF5 ΔIEC [DSS,DSS + Doxy]	0.126	ns
	2	ATF5 flox/flox versus ATF5 ΔIEC [DSS,DSS + Doxy]	0.311	ns
	3	ATF5 flox/flox versus ATF5 ΔIEC [DSS,DSS + Doxy]	0.071	ns
4J	1	ATF5 flox/flox versus ATF5 ΔIEC [Salmonella Typhimirium, Leptin + Salmonella Typhimirium]	0.177	ns
	2	ATF5 flox/flox versus ATF5 ΔIEC [Salmonella Typhimirium, Leptin + Salmonella Typhimirium]	0.549	ns
	3	ATF5 flox/flox versus ATF5 ΔIEC [Salmonella Typhimirium, Leptin + Salmonella Typhimirium]	0.338	ns
	1	ATF5 flox/flox versus ATF5 ΔIEC [Salmonella Typhimirium]	0.026	*
	2	ATF5 flox/flox versus ATF5 ΔIEC [Salmonella Typhimirium]	0.004	**
	3	ATF5 flox/flox versus ATF5 ΔIEC [Salmonella Typhimirium]	7E-04	***
	1	ATF5 flox/flox versus ATF5 ΔIEC [Leptin + Salmonella Typhimirium]	0.177	ns
	2	ATF5 flox/flox versus ATF5 ΔIEC [Leptin + Salmonella Typhimirium]	0.549	ns
	3	ATF5 flox/flox versus ATF5 ΔIEC [Leptin + Salmonella Typhimirium]	0.365	ns
	1	ATF5 flox/flox versus ATF5 flox/flox [Leptin + Salmonella Typhimirium,Salmonella Typhimirium]	0.065	ns
	2	ATF5 flox/flox versus ATF5 flox/flox [Leptin + Salmonella Typhimirium,Salmonella Typhimirium]	0.075	ns
	3	ATF5 flox/flox versus ATF5 flox/flox [Leptin + Salmonella Typhimirium,Salmonella Typhimirium]	0.063	ns
	1	ATF5 ΔIEC versus ATF5 ΔIEC [Leptin + Salmonella Typhimirium, Salmonella Typhimirium]	0.01	*
	2	ATF5 ΔIEC versus ATF5 ΔIEC [Leptin + Salmonella Typhimirium, Salmonella Typhimirium]	0.004	**
	3	ATF5 ΔIEC versus ATF5 ΔIEC [Leptin + Salmonella Typhimirium, Salmonella Typhimirium]	0.033	*
5I	1	ATF5 flox/flox versus ATF5 ΔIEC [Salmonella Typhimirium, CCK + Salmonella Typhimirium]	0.304	ns
	2	ATF5 flox/flox versus ATF5 ΔIEC [Salmonella Typhimirium, CCK + Salmonella Typhimirium]	0.273	ns
	3	ATF5 flox/flox versus ATF5 ΔIEC [Salmonella Typhimirium, CCK + Salmonella Typhimirium]	0.804	ns
	1	ATF5 flox/flox versus ATF5 ΔIEC [Salmonella Typhimirium]	0.023	*
	2	ATF5 flox/flox versus ATF5 ΔIEC [Salmonella Typhimirium]	0.003	**
	3	ATF5 flox/flox versus ATF5 ΔIEC [Salmonella Typhimirium]	0.038	*
	1	ATF5 flox/flox versus ATF5 ΔIEC [CCK + Salmonella Typhimirium]	0.647	ns
	2	ATF5 flox/flox versus ATF5 ΔIEC [CCK + Salmonella Typhimirium]	0.396	ns
	3	ATF5 flox/flox versus ATF5 ΔIEC [CCK + Salmonella Typhimirium]	0.243	ns
	1	ATF5 flox/flox versus ATF5 flox/flox [CCK + Salmonella Typhimirium,Salmonella Typhimirium]	0.064	ns
	2	ATF5 flox/flox versus ATF5 flox/flox [CCK + Salmonella Typhimirium,Salmonella Typhimirium]	0.085	ns
	3	ATF5 flox/flox versus ATF5 flox/flox [CCK + Salmonella Typhimirium,Salmonella Typhimirium]	0.078	ns
	1	ATF5 ΔIEC versus ATF5 ΔIEC [CCK + Salmonella Typhimirium, Salmonella Typhimirium]	0.048	*
	2	ATF5 ΔIEC versus ATF5 ΔIEC [CCK + Salmonella Typhimirium, Salmonella Typhimirium]	0.012	**
	3	ATF5 ΔIEC versus ATF5 ΔIEC [CCK + Salmonella Typhimirium, Salmonella Typhimirium]	0.034	*
6H	1	ATF5 flox/flox versus ATF5 ΔIEC [Salmonella Typhimirium, 2-DG + Salmonella Typhimirium]	0.564	ns
	2	ATF5 flox/flox versus ATF5 ΔIEC [Salmonella Typhimirium, 2-DG + Salmonella Typhimirium]	0.934	ns
	3	ATF5 flox/flox versus ATF5 ΔIEC [Salmonella Typhimirium, 2-DG + Salmonella Typhimirium]	0.651	ns
	1	ATF5 flox/flox versus ATF5 ΔIEC [Salmonella Typhimirium]	8E-04	***
	2	ATF5 flox/flox versus ATF5 ΔIEC [Salmonella Typhimirium]	4E-04	***
	3	ATF5 flox/flox versus ATF5 ΔIEC [Salmonella Typhimirium]	<.0001	****
	1	ATF5 flox/flox versus ATF5 ΔIEC [2DG + Salmonella Typhimirium]	0.333	ns
	2	ATF5 flox/flox versus ATF5 ΔIEC [2DG + Salmonella Typhimirium]	0.655	ns
	3	ATF5 flox/flox versus ATF5 ΔIEC [2DG + Salmonella Typhimirium]	0.564	ns
	1	ATF5 flox/flox versus ATF5 flox/flox [2DG + Salmonella Typhimirium,Salmonella Typhimirium]	0.409	ns
	2	ATF5 flox/flox versus ATF5 flox/flox [2DG + Salmonella Typhimirium,Salmonella Typhimirium]	0.09	ns
	3	ATF5 flox/flox versus ATF5 flox/flox [2DG + Salmonella Typhimirium,Salmonella Typhimirium]	0.062	ns
	1	ATF5 ΔIEC versus ATF5 ΔIEC [2DG + Salmonella Typhimirium, Salmonella Typhimirium]	0.001	**
	2	ATF5 ΔIEC versus ATF5 ΔIEC [2DG + Salmonella Typhimirium, Salmonella Typhimirium]	0.044	*
	3	ATF5 ΔIEC versus ATF5 ΔIEC [2DG + Salmonella Typhimirium, Salmonella Typhimirium]	0.031	*
6I	1	ATF5 flox/flox versus ATF5 ΔIEC [Citrobacter Rodentium, 2-DG + Citrobacter Rodentium]	0.655	ns
	2	ATF5 flox/flox versus ATF5 ΔIEC [Citrobacter Rodentium, 2-DG + Citrobacter Rodentium]	0.306	ns
	3	ATF5 flox/flox versus ATF5 ΔIEC [Citrobacter Rodentium, 2-DG + Citrobacter Rodentium]	0.141	ns
	1	ATF5 flox/flox versus ATF5 ΔIEC [Citrobacter Rodentium]	0.03	*
	2	ATF5 flox/flox versus ATF5 ΔIEC [Citrobacter Rodentium]	0.042	*
	3	ATF5 flox/flox versus ATF5 ΔIEC [Citrobacter Rodentium]	0.004	**
	1	ATF5 flox/flox versus ATF5 ΔIEC [2DG + Citrobacter Rodentium]	0.411	ns
	2	ATF5 flox/flox versus ATF5 ΔIEC [2DG + Citrobacter Rodentium]	0.187	ns
	3	ATF5 flox/flox versus ATF5 ΔIEC [2DG + Citrobacter Rodentium]	0.37	ns
	1	ATF5 flox/flox versus ATF5 flox/flox [2DG + Citrobacter Rodentium,Citrobacter Rodentium]	0.066	ns
	2	ATF5 flox/flox versus ATF5 flox/flox [2DG + Citrobacter Rodentium,Citrobacter Rodentium]	0.071	ns
	3	ATF5 flox/flox versus ATF5 flox/flox [2DG + Citrobacter Rodentium,Citrobacter Rodentium]	0.263	ns
	1	ATF5 ΔIEC versus ATF5 ΔIEC [2DG + Citrobacter Rodentium,Citrobacter Rodentium]	0.001	**
	2	ATF5 ΔIEC versus ATF5 ΔIEC [2DG + Citrobacter Rodentium,Citrobacter Rodentium]	0.038	*
	3	ATF5 ΔIEC versus ATF5 ΔIEC [2DG + Citrobacter Rodentium,Citrobacter Rodentium]	0.001	**
S2C	1	ATF5 flox/flox versus ATF5 ΔIEC [Salmonella Typhimirium]	0.032	*
	2	ATF5 flox/flox versus ATF5 ΔIEC [Salmonella Typhimirium]	0.012	*
	3	ATF5 flox/flox versus ATF5 ΔIEC [Salmonella Typhimirium]	0.043	*
S3E	1	ATF5 flox/flox versus ATF5 ΔIEC [Citrobacter Rodentium]	0.032	*
	2	ATF5 flox/flox versus ATF5 ΔIEC [Citrobacter Rodentium]	0.005	**
	3	ATF5 flox/flox versus ATF5 ΔIEC [Citrobacter Rodentium]	0.024	*
